# *Sarcandra glabra*: phytochemistry, pharmacological activities, and its role in mucosal immunity and digestive diseases

**DOI:** 10.3389/fimmu.2026.1851792

**Published:** 2026-07-07

**Authors:** Siyi Qian, Yue Zhang, Yining Guan, Jintao Chen, Jiaxing Cai, Yang Zheng, Xuewei Ye, Wenxiu Xin

**Affiliations:** 1 School of Pharmaceutical Sciences, Zhejiang Chinese Medical University, Hangzhou, China; 2 Key Laboratory of Artificial Organs and Computational Medicine in Zhejiang Province, Key Laboratory of Pollution Exposure and Health Intervention of Zhejiang Province, Shulan International Medical College, Zhejiang Shuren University, Hangzhou, China; 3 Department of Pharmacy, Zhejiang Cancer Hospital, Hangzhou, China; 4 Proteomics and Metabolomics Facility, Cornell University, Ithaca, NY, United States

**Keywords:** clinical application, digestive system disease, pharmacological action, research progress, *Sarcandra glabra*

## Abstract

The incidence of digestive system diseases has been increasing annually, highlighting the need for effective therapeutic agents. *Sarcandra glabra* (Thunb.) Nakai, a key Chinese herbal medicine, has gained attention for its potential in treating digestive disorders. The purpose of this review is to explore the research progress of *Sarcandra glabra* and its compound preparations in the treatment of digestive system diseases, so as to promote the further exploration of its pharmacological mechanism and the optimization of its clinical 2024 application. *Sarcandra glabra* contains a variety of chemical constituents, including sesquiterpenes, coumarins, flavonoids, organic acids, polysaccharides and volatile oils, which endow *Sarcandra glabra* with a wide range of pharmacological effects, such as antibacterial (against *Helicobacter pylori*, Shigella, Staphylococcus aureus), anti−inflammatory (via TLR4/NF−κB and MAPK pathways), gastroprotective (through mucosal repair, upregulation of tight junction proteins claudin−1 and occludin, and antioxidant activity), immunomodulatory (via Th17/Treg balance, secretory immunoglobulin A (SIgA) secretion, and dendritic cell activation), and anti−tumor (by inducing apoptosis, cell cycle arrest, and telomerase inhibition). Clinically, *S. glabra* and its various formulations (injections, tablets, granules, oral liquids) have been used for infectious diarrhea, gastritis, peptic ulcers, and as adjuvant therapy for nasopharyngeal, gastric, and colorectal cancers, showing improvements in clinical symptoms and quality of life. However, most clinical evidence is derived from small−scale, non−randomized, or uncontrolled studies. Short−term use is generally well tolerated, with mild gastrointestinal discomfort being the most common adverse event; toxicological studies indicate low acute toxicity and no mutagenicity, but long−term safety and chronic toxicity data are lacking. Future research should prioritize high−quality randomized controlled trials, systematic pharmacovigilance, and mechanistic studies focusing on gastrointestinal mucosal immunity and gut microbiota modulation. In summary, *Sarcandra glabra* exhibits multiple pharmacological activities relevant to digestive system diseases, including anti-inflammatory, antibacterial, gastroprotective, and immunomodulatory effects. These properties suggest potential therapeutic value, although current evidence is primarily preclinical or derived from small-scale clinical studies. Further high-quality randomized controlled trials and systematic safety evaluations are needed to confirm its efficacy and establish its role in clinical practice. Through systematic and in-depth research and development, *Sarcandra glabra* is expected to bring treatment options and hope to more patients.

## 1 Introduction

*Sarcandra glabra* (Thunb.) Nakai *(S. glabra)*, a member of the family Chloranthaceae, is widely used in Traditional Chinese Medicine (TCM). It is known by several vernacular names, including Zhongjiefeng (ZJF),Caoshanhu and Jiujie Tea, which have been recorded in various historical texts. The plant was first documented in the classical text Essentials of Herbal Medicine (1, 2). The name “Caoshanhu” originated from the Flora of Yunnan, while the alias “Jiujie Tea” was first recorded in the History of Runan Garden during the Ming Dynasty. Its medicinal properties were first described in the Essentials of Raw Herbal Medicine from the Qing Dynasty. Furthermore, the Records of Collecting Herbs in the South of the Five Ridges noted that “Guanyin Tea, also known as Jiujie Tea, is astringent in taste and neutral in nature, and can be decocted to reduce fever.” The Mountain Herbal Guide further mentioned that “Jiujie Tea is wet in taste and neutral in nature. It can be decocted to reduce fever, dispel wind, and remove dampness” (3).

In clinical TCM practice, the whole plant or root tuber of *S. glabra* is commonly used for medicinal purposes. It is characterized by pungent and bitter flavors with a mild property, is traditionally utilized for antibacterial and anti-inflammatory purposes, cooling blood, clearing heat and detoxification, expelling wind and dredging collaterals, promoting blood circulation and resolving masses (1). Modern pharmacological studies have shown that *S. glabra* exhibits a wide range of biological activities, including analgesic, antibacterial, anti-inflammatory, gastroprotective, anti-tumor effects. In addition, it has demonstrated immunomodulatory properties, further highlighting its medicinal value and validating its status as a representative Chinese medicinal herb.

Based on the diverse therapeutic effects, numerous Zhongjiefeng preparations based on *S. glabra* have been developed and are now widely used in clinical settings. Common Zhongjiefeng preparations formulations in clinic include Zhongjiefeng injection, Zhongjiefeng Sanqing Granules, Zhongjiefeng tablets, Xuekang Oral Liquid, Caoshanhu Buccal Tablets, Compound Zhongjiefeng Qingyan Mixture, etc. These preparations are widely recognized for their safety and efficacy, and broad therapeutic applications. Clinically, they are used for pain relief, treatment of gastric ulcer, tumor inhibition, and as adjuvant therapies.

Digestive system diseases, including inflammatory bowel disease, functional gastrointestinal diseases, and gastrointestinal tumors, are often chronic, recurrent, and associated with multiple complications, which pose considerable challenges to clinical management. With advances in research, the significant role of *S. glabra* and its related formulations in the prevention and treatment of digestive system diseases has attracted growing attention. However, a comprehensive review of the available findings in this field is still lacking. To address this gap, we searched the PubMed, CNKI, and Web of Science databases using the keywords “*Sarcandra glabra*”, “Zhongjiefeng”, “Caoshanhu”, combined with “phytochemistry”, “pharmacology”, “digestive system diseases”, “mucosal immunity”, and “clinical application”. Articles published up to March 2026 were considered with priority given to original research, clinical trials, and recent reviews. Based on this search, this article provides a narrative review of the phytochemical components, pharmacological activities, and clinical applications of *S. glabra* and its related preparations in digestive system diseases, aiming to offer a theoretical foundation for future research and to promote the rational clinical use and innovative development of this herb in this therapeutic area.

## 2 Main chemical constituents of *Sarcandra glabra*

*Sarcandra glabra* (Thunb.) Nakai (*S. glabra*) contains a wide range of bioactive compounds, among which sesquiterpenes, coumarins, flavonoids and organic acids, and polysaccharides are the most representative. Additionally, the plant is rich in volatile oils and trace elements. These components are believed to collectively contribute to the plant’s multiple pharmacological effects, including anti-inflammatory, analgesic, antimicrobial and antitumor activities.

### 2.1 Sesquiterpenes

Sesquiterpenoids are among the primary constituents of *S. glabra* and are considered key indicators of its chemical profile (**Table 1**). These compounds have been reported to exhibit multiple biological activities, including antitumor, antibacterial, anti-inflammatory and antispasmodic effects. The diversity and activity of sesquiterpenoids make them important candidates for further pharmacological exploration. Sesquiterpenoids, especially chloranthalactones A-G, are the dominant anti−inflammatory components. Several sarcaglabosides A-H exhibit hepatoprotective activity. The presence of atractylenolides II-IV suggests additional anti−inflammatory and antitumor potential. Notably, sarglaperoxide A shows dual antibacterial and anti−inflammatory effects.

**Table 1:** Sesquiterpenoids in *Sarcandra glabra*.

No.	Name	Chemical formula	Relative molecular mass	Activity	Literature
1	Chloranthalactone A	C_15_H_16_O_2_	228.29	Anti-inflammatory	(3)
2	Chloranthalactone B	C_15_H_16_O_3_	244.28	Anti-inflammatory	(4–6)
3	Chloranthalactone C	C_17_H_20_O_4_	288.34	Anti-inflammatory	(7)
4	Chloranthalactone E	C_15_H_18_O_4_	262.30	Anti-inflammatory	(8)
5	Chloranthalactone F	C_15_H_16_O_3_	244.29	Anti-inflammatory	(4)
6	Chloranthalactone G	C_15_H_16_O_3_	244.29	Anti-inflammatory	(3)
7	Chloranoside A	C_21_H_28_O_9_	424.44	Hepatoprotective	(9)
8	Chloranthalactone E-8-O-β-D-glucopyranosige	C_21_H_28_O_9_	424.44	Hepatoprotective	(9)
9	Sarcaglaboside A	C_21_H_30_O_8_	410.46	Hepatoprotective	(9)
10	Sarcaglaboside B	C_21_H_28_O_8_	408.44	Hepatoprotective	(9)
11	Sarcaglaboside C	C_21_H_30_O_8_	410.46	Hepatoprotective	(9)
12	Sarcaglaboside D	C_26_H_38_O_12_	542.57	Hepatoprotective	(9)
13	Sarcaglaboside E	C_26_H_38_O_12_	542.57	Hepatoprotective	(9)
14	Sarcaglaboside F	C_21_H_28_O_10_	440.44	Anti-inflammatory	(4)
15	Sarcaglaboside G	C_21_H_30_O_9_	426.46	Anti-inflammatory	(4)
16	Sarcaglaboside H	C_21_H_32_O_9_	428.47	Anti-inflammatory	(4)
17	Istanbulin A	C_15_H_20_O_4_	264.32	Antibacterial	(10)
18	Istanbulin B	C_15_H_20_O_3_	248.32	Antibacterial	(7)
19	Atractylenolide II	C_15_H_20_O_2_	232.32	Anti-inflammatory	(11–13)
20	Atractylenolide III	C_15_H_20_O_3_	248.32	Anti-inflammatory Antitumor	(12, 13)
21	Atractylenolide IV	C_17_H_22_O_5_	306.35	Anti-inflammatory Antitumor	(12, 13)
22	Sarcandralactone A	C_15_H_18_O_3_	246.30	Anti-inflammatory	(14)
23	Sarcandralactone B	C_15_H_18_O_3_	246.30	Anti-inflammatory	(15)
24	Sarglaperoxide A	C_15_H_16_O_3_	244.29	Antibacterial, Anti-inflammatory	(16)

### 2.2 Coumarins

Coumarins are another major class of active constituents in *S. glabra* (**Table 2**). Among them, isofraxitin is regarded as a key representative compound, and is officially listed for quality control in the *Chinese Pharmacopoeia* (2020 edition) (30). Isofraxidin exerts its anti-inflammatory effects through modulation of MAPK and NF-κB signaling pathway, including the regulation of inflammatory factor TNF-α production (31). Notably, isofraxidin also demonstrates significant antibacterial and anticancer effects properties, with relatively high abundance in *S. glabra*, reinforcing its potential as both a therapeutic agent and a quality control marker. Isofraxidin is one of the more extensively studied coumarins, acting via MAPK/NF−κB pathways. Its derivatives (e.g., 3,3′−biisofraxidin) also possess anti−inflammatory activity. Other coumarins such as esculetin and scopoletin contribute to antibacterial and hepatoprotective effects.

**Table 2:** Coumarins in *Sarcandra glabra*.

No.	Name	Chemical formula	Relative molecular mass	Activity	Literature
1	Coumarin	C_9_H_6_O_2_	146.14	Antibacterial	(17)
2	Esculetin	C_9_H_6_O_4_	178.14	Anti-inflammatory Antitumor Antibacterial	(18)
3	Scoparone	C_11_H_10_O_4_	206.19	Anti-inflammatory Hepatoprotective	(19–21)
4	Isofraxidin	C_11_H_10_O_5_	222.19	Anti-inflammatory	(22, 23)
5	Isofraxidin-7-O-β-D-glucopyranoside	C_17_H_20_O_10_	384.33	Anti-inflammatory	(24)
6	3,3′-biisofraxidin	C_22_H_18_O_10_	442.37	Anti-inflammatory	(23)
7	4,4′-biisofraxidin	C_22_H_18_O_10_	442.37	Anti-inflammatory	(23)
8	Fraxidin	C_11_H_10_O_5_	222.19	Antibacterial	(24)
9	Fraxetin	C_10_H_8_O_5_	208.17	Anti-inflammatory Hepatoprotective	(25)
10	Fraxin	C_16_H_18_O_10_	370.31	Anti-inflammatory Hepatoprotective	(26)
11	Scopoletin	C_10_H_8_O_4_	192.17	Anti-inflammatory Antitumor Antibacterial	(27)
12	Scopolin	C_16_H_18_O_9_	354.31	Anti-inflammatory	(28)
13	Eleutheroside B1	C_17_H_20_O_10_	384.33	Anti-inflammatory Anti-viral	(29)

### 2.3 Flavonoids

*Sarcandra glabra* is rich in total flavonoids (**Table 3**), especially flavanones and dihydrochalcones. These flavonoids exhibit various biological activities, with immunoregulatory and antioxidant effects being particularly noteworthy. For example, low doses of flavonoids enhance immunity in immunocompromised individuals, whereas higher doses may help suppress excessive immune responses. Such bidirectional immunomodulatory effects may also confer anti−fatigue, anti-aging, and anti-infective benefits, resembling those of ginseng.

**Table 3:** Flavonoids in *Sarcandra glabra*.

No.	Name	Chemical formula	Relative molecular mass	Activity	Literature
1	Quercetin	C_15_H_10_O_7_	302.24	Anti-inflammatory	(32)
2	Quercetin 3-O-β-D-glucuronopyranoside	C_21_H_20_O_11_	448.38	Anti-inflammatory	(33)
3	Glabraoside A	C_30_H_30_O_13_	598.55	Antioxidant	(34)
4	Glabraoside B	C_30_H_30_O_13_	598.55	Antioxidant	(34)
5	Catechin 3-O-α-L-rhamnopyranoside	C_21_H_24_O_10_	436.41	Antioxidant	(35)
6	2,4,4-Trihydroxy-chalcone	C_15_H_12_O_4_	256.25	Antioxidant	(36)
7	2’,4-Dihydroxy-4’,6’-dimethoxychalcone	C_17_H_16_O_5_	300.31	Anti-inflammatory	(37)
8	5-Hydroxy-7- methoxyflavanone	C_16_H_14_O_4_	270.28	Anti-inflammatory	(3)
9	Kaempferol	C_15_H_10_O_6_	286.24	Anti-inflammatory Antioxidant	(38)
10	Kaempferol-3-O-β-D-glucuronide	C_21_H_18_O_12_	462.36	Anti-inflammatory	(39)
11	Neoastilbin	C_21_H_22_O_11_	450.39	Anti-inflammatory	(40)
12	Pinostrobin	C_16_H_14_O_4_	270.28	Anti-inflammatory Antioxidant	(41)

In addition, *S. glabra* flavonoid extracts display excellent antioxidant activity in a dose-dependent manner. Their DPPH radical scavenging capacity has shown to exceed that of vitamin C, and their hydroxyl radical scavenging capacity is comparable to vitamin C (42). Flavonoids in *S. glabra* are diverse, including flavanones and dihydrochalcones. Quercetin and kaempferol derivatives are primarily anti−inflammatory, while glabraosides A/B and catechin rhamnoside are notable for antioxidant activity. Pinostrobin exhibits both anti−inflammatory and antioxidant properties.

### 2.4 Organic acids

Organic acids in *S. glabr*a contribute significantly to its antibacterial, anti-inflammatory, antiviral and other biological activities (**Table 4**). These compounds include both fatty acids and phenolic acids, such as fumaric acid, chlorogenic acid, and caffeic acid. The ethanol extract of the whole plant exhibits protective effects against hydroxyl radical-induced oxidative damage in mesenchymal stem cells, mainly attributed to its caffeic acid derivatives. Among them, rosmarinic acid, is notable for its ability to scavenge reactive oxygen species both directly and indirectly, highlighting its potent antioxidant capability (55). Rosmarinic acid is a key antioxidant that scavenges ROS directly and indirectly. Chlorogenic and caffeic acids contribute to anti−inflammatory and antioxidant effects. Several phenolic acids (e.g., ferulic, vanillic) provide additional antioxidant support, while fumaric and shikimic acids show antibacterial activity.

**Table 4:** Organic acids in *Sarcandra glabra*.

No.	Name	Chemical formula	Relative molecular mass	Activity	Literature
1	Fumaric acid	C_4_H_4_O_4_	116.07	Antioxidant	(43)
2	Shikimic Acid	C_7_H_10_O_5_	174.15	Antibacterial	(44)
3	Quinic acid	C_7_H_12_O_6_	192.17	Antibacterial Antioxidant	(45)
4	Ferulic Acid	C_10_H_10_O_4_	194.18	Antioxidant	(46)
5	Vanillic acid	C_8_H_8_O_4_	168.15	Anti-inflammatory Antioxidant	(47)
6	3,4-dihydroxybenzoic acid	C_7_H_6_O_4_	154.12	Antioxidant	(47)
7	Chlorogenic acid	C_16_H_18_O_9_	354.31	Antioxidant	(48)
8	Caffeic acid	C_9_H_8_O_4_	180.16	Anti-inflammatory Antioxidant	(49)
9	Palmitic acid	C_16_H_32_O_2_	256.42	Anti-inflammatory	(50)
10	Pentadecanoic acid	C_15_H_30_O_2_	242.40	Anti-inflammatory	(51)
11	Dibutyl phthalate	C_16_H_22_O_4_	278.34	Antioxidant	(52)
12	Rosmarinic acid	C_18_H_16_O_8_	360.31	Anti-inflammatory	(53, 54)

### 2.5 Polysaccharides

Polysaccharides isolated from *S. glabr*a via water extraction and alcohol precipitation have demonstrated promising immunoregulatory effects. For instance, *S. glabr*a polysaccharides modulate cytokine production in RAW264.7 cells by promoting membrane protein-related immune molecules (56). Liu et al. (57) found that purified acidic polysaccharide from *S. glabr*a can enhance the antitumor effects of various cancer vaccines by activating dendritic cells through the TLR4 pathway, thereby strengthening both humoral and cellular immune responses. Moreover, these polysaccharides show strong antioxidant activity. For example, the homopolysaccharide SGP-1 effectively scavenges hydroxyl, superoxide, DPPH and ABTS radicals in a dose-dependent manner (58).

### 2.6 Other compounds

In addition to the above constituents, *S. glabra* also contains a large amount of volatile oils and various trace elements. Huang et al. (59)analyzed the volatile oil composition using Gas Chromatography-Mass Spectrometry (GC-MS). They identified over 80 chromatographic peaks and characterized 38 individual compounds. The main components included elemene (14.41%), 1-p-menthene-8-acetate (13.71%), eremrene (18.55%) and β-ocimene-X (6.88%).Furthermore, Yang et al. (60) identified 12 essential trace elements in *S. glabra*, including calcium (Ca), magnesium (Mg), iron (Fe), zinc (Zn), and copper (Cu), using ultraviolet-visible spectrophotometry, atomic absorption spectrometry and X-ray fluorescence analysis. These elements may contribute to its pharmacodynamic synergy and overall therapeutic potential.

**Figure 1:**
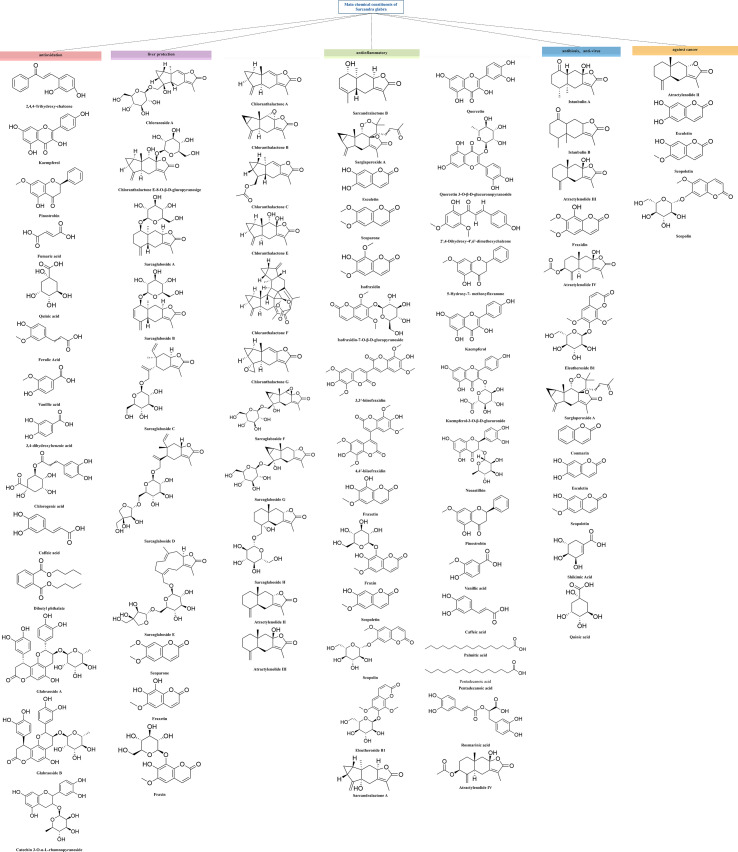
Main chemical constituents of *Sarcandra glabra* and their associated pharmacological activities. The figure presents the chemical structures of representative bioactive compounds isolated from *Sarcandra glabra*, organized according to their primary pharmacological properties into five categories: antioxidant (pink panel), liver protection (purple panel), anti-inflammatory (green panel), antibiotic and antiviral (blue panel), and anticancer (orange panel). Each structure is labeled with its corresponding compound name.

Together, these diverse chemical constituents form the pharmacological foundation of *S. glabra*, supporting its multifunctional therapeutic roles in traditional and modern medicine. **Figure 1** presents the structural formulas of the main chemical constituents of *Sarcandra glabra*, classified according to their primary pharmacological activities into five major categories: antioxidation, liver protection, anti−inflammatory, antibacterial/antiviral, and anti−cancer. Among these, sesquiterpenes (e.g., chloranthalactones) and coumarins (e.g., isofraxidin) are frequently studied bioactive classes, while flavonoids and organic acids contribute to antioxidant and anti−inflammatory activities. Polysaccharides, although less abundant, play key roles in immunomodulation. The structural diversity of these compounds forms the chemical basis for the multi−target pharmacological effects discussed in later sections.

## 3 Extraction technologies of *Sarcandra glabra*: traditional separation and modern techniques

### 3.1 Traditional extraction and separation techniques for *Sarcandra glabra*

Traditional extraction and separation of *S. glabra* primarily rely on solvent extraction, column chromatography, and the water decoction method described in the Chinese Pharmacopoeia. Solvent extraction, one of the most fundamental methods for extracting *S. glabra*, typically employs ethanol, methanol, or water as the extraction solvent. Studies have shown that reflux extraction with 70% ethanol for 6 hours yields a total flavonoid content of 6.3%, while employing a water-alcohol precipitation method (initial dissolution in water followed by ethanol precipitation) can increase the total flavonoid content to 7.3%. Column chromatography is widely used to isolate various active components from *S. glabra*. Silica gel column chromatography is the most commonly used stationary phase, and gradient elution with petroleum ether and ethyl acetate enables effective separation of components such as sesquiterpenes and flavonoids. The water decoction method, recorded in the 2000 edition of the Chinese Pharmacopoeia, involves boiling chopped *S. glabra* in water three times (1 hour each), combining the decoctions, filtering, and concentrating into a dry extract. Although these traditional methods are operationally simple, they suffer from drawbacks such as low extraction efficiency and potential solvent residues.

### 3.2 Modern analytical techniques for *Sarcandra glabra*

In recent years, researchers have focused on developing novel extraction processes and technologies for *S. glabra*. Modern techniques such as ultrasonic-assisted extraction and supercritical fluid extraction (SFE) have been applied to its extraction and separation. Ultrasonic extraction utilizes cavitation to accelerate the dissolution of active components, offering advantages like shorter extraction time, higher yield, and no requirement for heating. Supercritical fluid extraction is particularly suitable for heat-sensitive components and overcomes the significant loss of active ingredients often associated with traditional extraction methods. Shao (K3r) established a UPLC-LTQ-Orbitrap dynamic profiling method using a Waters CORTECS C18 column with gradient elution (acetonitrile-0.1% formic acid aqueous solution) and a mass spectrometry resolution of 30,000. An OPLS-DA model was also integrated for data analysis. This approach identified 31 compound components in *S. glabra*, including organic acids, flavonoids, coumarins, and sesquiterpenes, and clarified compositional differences across various growth stages. The UPLC-LTQ-Orbitrap technique showed a threefold increase in sensitivity compared to traditional HPLC methods. It enables simultaneous quantification of multiple components and is becoming the mainstream technology for component analysis of *S. glabra*. These modern analytical techniques not only enhance extraction efficiency but also improve the purity and stability of *S. glabra* components.

## 4 Pharmacological effects of *Sarcandra glabra* on the digestive system

### 4.1 Antibacterial activity

Preclinical and clinical studies have demonstrated that *S. glabra* exhibits broad-spectrum antibacterial property, with particularly prominent effects observed in its leaves, and fresh rhizomes, which show higher potency compared to dried samples (61). Experimental evidence has confirmed its efficacy against various bacterial strains, including *Staphylococcus aureus (S. aureus)*, *Shigella dysenteriae*, *Escherichia coli (E. coli)*, *Pseudomonas aeruginosa (P. aeruginosa)*, *Salmonella typhi*, and *Paratyphi A*. Notably, *S. glabra* shows strong inhibitory activity against *S. aureus* and Shigella dysenteriae type I *in vitro*. Furthermore, one study reported that it reduced mortality in a mouse model of S. aureus bacteremia (62). Further investigations revealed strong inhibitory activity against antibiotic-resistant strains, such as *S. aureus* resistant strains, *Shigella boydii* C-2, *Salmonella typhi* H901 and *Paratyphoid A*. Additionally, *S. glabra* demonstrated varying degrees of inhibition toward *Streptococcus haemolyticus*, *Shigella flexner*i type III, *E. coli* and *P. aeruginosa* (63). However, the original study did not provide quantitative data such as inhibition zone diameters or minimum inhibitory concentration (MIC) values; therefore, the potency of these effects cannot be compared. Bioactive compounds such as sarglaperoxide A, a sesquiterpene isolated from ethanol extracts of *S. glabra*, significantly inhibited the growth of S. aureus at 25 μg/mL, achieving a 64.5% inhibition rate (16). However, the original study did not report a standard MIC value; the reported concentration represents a single tested dose rather than the MIC. Volatile oils extracts from *S. glabra* also exhibited anti-fungal activity against *Candida albicans* (61). Moreover, Total flavonoids from *S. glabra* showed bacteriostatic rates of 47.03% and 27.78% against *E. coli* and *S. aureus*, respectively (64), MIC values were not provided in that study. The water extract of *S. glabra* was found to compromise the integrity of the Helicobacter pylori (*H. pylori*) outer membrane, thereby increasing the permeability of glucose and aspartate aminotransferase. This resulted in mild bacteriostatic effects against drug-resistant *H. pylori* strains. The original study did not report specific MIC values. Notably, synergistic antibacterial effects were observed when combined with commonly used antibiotics such as amoxicillin, clarithromycin, metronidazole, levofloxacin, tetracycline and furazolidone (65). *In vivo*, crystalline compound A extracted from the whole herb exhibited bacteriostatic activity (66). Organic acids such as fumaric acid and succinic acid also show moderate to high inhibitory effects against pathogens including *Streptococcus* spp. and influenza viruses (61), but quantitative MIC data are lacking in the available literature. Comparative studies indicated that Zhongjiefeng tablets exhibited greater inhibition of *S. aureus* and *E. coli* than Shuanghuanglian and Chaiyin Oral Liquid and showed comparable efficacy to Shuanghuanglian Oral Liquid in inhibiting P. aeruginosa (67). However, quantitative MIC data are generally lacking in these studies. Future investigations should include MIC determination to facilitate cross−comparison of antibacterial potency.

### 4.2 Antiviral activity

A clinical study (68) reported that a single−herb injectable formulation of *S. glabra* (Zhongjiefeng injection) had a therapeutic effect against viral enteritis in children, based on observed symptom improvement. Huang Guohui treated 42 cases of infantile rotavirus enteritis with Zhongjiefeng injection, while the control group received ribavirin. The results showed a total response rate of 100% and a marked response rate of 95.2%. However, the original study did not provide quantitative outcome measures such as response rates or statistical comparisons with a control group.

In addition, multi-component TCM formulations contains *S. glabra* has shown combined antiviral, antibacterial and gastrointestinal regulatory effects. These preparations improve intestinal blood circulation, enhance water reabsorption and shorten the disease course. They are particularly beneficial for patients with early-stage mild and moderate dehydration caused by rotavirus or enterotoxigenic *E. coli* infection, especially under limited primary care conditions where stool culture are unavailable (69).

### 4.3 Anti-inflammatory effects in digestive system

Multiple extracts of *S. glabra*, including coumarins, polysaccharides, and isofraxidin, have been reported to exhibit anti−inflammatory activity in various *in vitro* and *in vivo* models (70). The underlying mechanism involves the regulation of key inflammatory signaling pathways, including Toll-like receptor 4 (TLR4) and nuclear factor-κB (NF-κB), as well as the downregulation of pro-inflammatory mediators such as nitric oxide (NO), interleukin-6 (IL-6), and tumor necrosis factor-alpha (TNF-α) (71). Notably, TLR4 activation is known to induce the production of TNF-α, IL-6, and type I interferons through MyD88- and TRIF-dependent cascades, and its dysregulation has been implicated in various inflammatory and autoimmune diseases affecting the digestive system (72). A similar anti−inflammatory mechanism has been documented in flavonoids from Trollius species, where orientin and vitexin suppress NF−κB activation and reduce IL−6 and TNF−α production (73–75). This suggests that some structurally diverse natural products may share common inflammatory pathways, although further comparative studies are needed. Studies have shown that lipopolysaccharide (LPS) significantly increases the phosphorylation of MAPK and NF−κB as well as the serum levels of TNF−α and IL−6, whereas these levels are markedly reduced upon co−treatment with *S. glabra* injection and lycopene (76).

Crystalline compound A derived from *S. glabra* has been used clinically in the form of 10 mg/mL injection, showing good therapeutic effects on suppurative tonsillitis and enteritis (66). Moreover, Zhongjiefeng tablets showed inhibitory effects in multiple inflammation models, including mouse ear edema, rat paw edema, and cotton pellet-induced granulomas (62).

A novel dicoumarin compound, 3,5-dihydroxy-7-O-α-L-rhamnopyranosyl-2H-chromen-2-one, was isolated from *S. glabr*a. This compound significantly suppressed nitric oxide (NO) production in macrophages by inhibiting the expression of inducible nitric oxide synthase (iNOS) mRNA, suggesting its potential as an anti−inflammatory agent (77). *S. glabra* polysaccharides act through a similar mechanism by enhancing the expression of macrophage−related immune molecules and immune factor RNAs, and by balancing pro−inflammatory and anti−inflammatory cytokine expression in macrophages, indicating their potential for regulating immune homeostasis (70).

Treatment with 100 ng/mL LPS induced IL−1 expression in a vascular endothelial cell line. MTT assay results showed that after 24 h of LPS stimulation, intervention with 3% *S. glabra* inhibited LPS−induced IL−1 expression in endothelial cells but had no significant effect on cell viability. These findings suggest that *S. glabra* may exert its therapeutic effect by stabilizing the metabolic activity of endothelial cells, regulating the secretory dysfunction of vascular endothelial cells caused by endotoxic shock, suppressing the excessive release of the inflammatory cytokine IL−1, and thereby blocking the inflammatory cascade (78).

### 4.4 Gastroprotective and anti-ulcer effects

As the important natural protective barrier of gastric wall, gastric mucosa maintains the dynamic homeostasis between injury and repair processes (79). Oxidative stress, particularly the accumulation of reactive oxygen species (ROS), is a major contributor to gastric mucosal injury. Phytochemicals in *S. glabra*, such as rosmarinic acid and astilbin, exhibit strong antioxidant activities by directly or indirectly scavenging ROS, thereby mitigating oxidative stress and inflammatory damage to tissues.

Clinical and experimental studies have demonstrated that *S. glabra* possesses significant gastroprotective and anti-ulcer properties. A study (80) involving 300 patients with raised erosive gastritis treated with *S. glabra* soft capsules reported that after completion of treatment and a 3−month follow−up, the recurrence rate in the *S. glabra* treatment group was 16.0%, compared with 34.0% in the control group, and the difference between the two groups was statistically significant. These findings suggest that *S. glabra* may ameliorate gastric mucosal inflammation, reverse dysplasia and intestinal metaplasia, protect and repair the gastric mucosa, inhibit ulcer formation, and suppress gastrointestinal hormones under pathological conditions, thereby promoting the resolution and absorption of inflammation. In addition, *S. glabra* may exert its effects through antibacterial activity, helping to control gastric bacteria, facilitate the resolution of local mucosal inflammation, alleviate edema, and gradually restore normal circulatory and secretory functions.

Furthermore, Zhongjiefeng injection not only inactivates L615 cells and directly suppresses cancer cells but also improves energy metabolism and lowers tumor toxin levels. In addition, it protects and repairs the gastric mucosa, exerts a marked astringent effect on bleeding ulcers, and facilitates rapid ulcer healing (81).

*S. glabra* extracts produced therapeutic outcomes comparable to aluminum sulfate by forming a protective film over the mucosa through interactions with mucosal proteins and inhibition of pepsin-mediated self-digestion in the treatment of gastric ulcer and erosive gastritis (82).

Collectively, these findings suggest that the gastroprotective effects of *S. glabra* are mediated through multiple mechanisms, including mucosal barrier repair, anti- inflammatory activity, antioxidant action, and potential modulation of gastrointestinal secretions.

### 4.5 Anti-tumor effects and mechanism

Malignant tumors of the digestive system, including gastric, liver and colorectal cancer, pose a major threat to global health. Several pharmacological and clinical studies have reported anti−tumor activity of *S. glabra* across multiple tumor types. However, it should be noted that many of these studies lack detailed quantitative data, and the evidence remains largely preclinical. Its mechanisms involve inhibition of cell cycle progression, induction of apoptosis, cell cycle arrest, regulation of oxidative stress, suppression of telomerase activity, and modulation of immune responses. Extracts of *S. glabra* as well as compound preparations such as Zhongjiefeng injection and Zhongjiefeng Dispersible Tablets, has shown therapeutic potential in both monotherapy and combination with chemotherapeutic agents (83, 84).

#### 4.5.1 Anti-nasopharyngeal carcinoma activity

*In vivo*, telomerase activity in CNE1 and CNE2 xenograft tumor cells was significantly lower in *S. glabra*−treated nude mice than in the control group (P < 0.01), indicating marked inhibition of telomerase activity. The extract reduced tumor growth in nude mice bearing CNE−1 and CNE−2 xenografts, with inhibition rates of 40.8% and 46.8%, respectively. These effects may be mediated through the induction of apoptosis and the suppression of telomerase activity (85). *In vitro*, flow cytometry results showed that as the apoptosis rate increased, the proliferation index (PI) decreased. *S. glabra* extract significantly inhibited the proliferation of CNE1, CNE2, TWO3, and C666−1 cells, and the inhibition increased with higher drug concentration and longer treatment duration, demonstrating dose−dependent and time−dependent relationships. After *S. glabra* treatment, the proportion of cells in G1 phase increased by 11.5%, 18.0%, 15.9%, and 12.6%, respectively, compared with the control group, while the proportion of cells in S phase decreased by 1.0%, 10.6%, 7.7%, and 10.1%, respectively, and the proportion in G2/M phase decreased by 10.5%, 7.4%, 8.2%, and 2.5%, respectively. These findings indicate that DNA synthesis was inhibited and that cells were arrested in the G0/G1 phase, with concomitant reductions in the S and G2/M phase populations, thereby suppressing tumor growth (86).

Further studies indicated that *S. glabra* exhibits radiosensitizing properties, enhancing ROS-mediated damage and selectively increasing the susceptibility of CNE 2 cells to radiation while promoting superoxide generation and hydroxyl radical scavenging (87).Clinically, co-administration of Zhongjiefeng decoction during radiotherapy has been reported to alleviate radiation-induced xerostomia and mucosal injury, with protective effects on healthy tissues (88). Additionally, *S. glabra* has been shown to inhibit the expression of Epstein−Barr virus (EBV) antigens, suggesting potential for prophylactic use in EBV−associated NPC (89).

#### 4.5.2 Anti-gastric cancer activity

*In vitro* studies have demonstrated that Zhongjiefeng injection effectively inhibits the proliferation of human gastric cancer SGC-7901 cells by promoting early apoptosis and arresting the cell cycle in the S phase (90). *In vivo*, tumor growth inhibition was also confirmed in the xenograft models. Similar inhibitory effects were observed in BGC-823 and MGC-803 gastric cancer cell lines, with dose-dependent cytotoxicity (91). Among the active compounds, 3,3′-biisofraxidin (a dimer of isofraxidin)exerts antiproliferative effects through mitochondria-mediated apoptosis pathway (92). Eleutheroside B1, a 7-position glycosylation derivative of isofraxidin, also showed strong cytotoxicity toward BGC-823 cells (93). Combination therapy using Zhongjiefeng injection with 5-fluorouracil (5-Fu) enhances chemotherapy efficacy (94). The immunomodulatory effect of *S. glabra* is also implicated, as evidenced by increased natural killer (NK) cells activity and TNF production in tumor-bearing mice. Additionally, total flavonoids from *S. glabra* were reported to inhibit Ribonucleic Acid (RNA) and Deoxyribonucleic Acid (DNA) synthesis in cancer cells, reduce the risk of ulcer carcinogenesis, and promote immune−mediated tumor suppression (95).

#### 4.5.3 Anti-hepatoma activity

*In vivo* and *in vitro* models of hepatocellular carcinoma have shown that *S. glabra* extracts significantly inhibit tumor cell proliferation and promote immune system activation. Zhongjiefeng Compound inhibited human hepatoma HepG2 cells growth in a dose-dependent manner and reduced telomerase activity (96). The mechanism may involve regulation of PTEN, p53, Bcl-2/Bax ratios, with Bax upregulation promoting apoptosis (97).In comparative studies, Zhongjiefeng injection exhibited inhibitory effects on Bel-7404 cells, and when combined with 5-Fu, synergistically inhibited tumor growth in the H22 mouse model while reducing 5-Fu-induced toxicity (98).

When *Sarcandra glabra* volatile oil (SGE) was combined with cyclophosphamide (CTX), it exhibited synergistic anti−tumor effects against both H22 hepatoma and S180 sarcoma to varying degrees. The combination of CTX (10 mg/kg or 15 mg/kg) with intravenous SGE (36.6 mg/kg) resulted in higher tumor inhibition rates against H22 and S180 in mice compared with CTX alone at the corresponding doses (P < 0.05). Similarly, CTX (20 mg/kg) combined with oral SGE (73.2 mg/kg) produced a higher tumor inhibition rate against H22 than CTX alone (P < 0.05). SGE also significantly antagonized the CTX−induced decrease in platelet and white blood cell counts (P < 0.05 to 0.01). These results indicate that SGE enhances the anti−tumor activity of CTX while reducing its toxicity (99).

#### 4.5.4 Anti-colorectal cancer activity

*In vitro* experiments showed that treatment of HCT-29 colon cancer cells with Zhongjiefeng injection at a concentration of 25 mg/ml for 4 days inhibited cell growth in a concentration-and time−dependent manner. At concentrations of 3.125, 6.25, 12.5, 25, and 50 μg/ml, the growth inhibition rate of human colon cancer HCT-29 cells increased with increasing drug concentration, with an half−maximal inhibitory concentration (IC_50_) value of 60.00 μg/ml, indicating that Zhongjiefeng exhibits strong cytotoxic effects against HCT-29 colon cancer cells (91). Zhongjiefeng injection inhibited the proliferation of human colon cancer HCT-8 cells, with an IC_50_ value of 52.39 mg/mL. At concentrations of 3.125, 6.25, and 12.5 mg/mL, the combination of Zhongjiefeng injection with doxorubicin showed additive inhibitory effects on HCT-8 cells *in vitro*. At concentrations of 25 and 50 mg/mL, the combination exhibited synergistic inhibitory effects. These findings indicate that Zhongjiefeng injection alone has a certain inhibitory effect on the growth of HCT-8 cells, and its combination with doxorubicin produces additive or synergistic inhibitory effects, with particularly strong synergy observed at higher concentrations of Zhongjiefeng injection (100).

### 4.6 Immunomodulatory effect

#### 4.6.1 Mechanisms of action on gastrointestinal mucosal immunity

The gastrointestinal mucosa represents the largest interface between the host and the external environment, and its immune homeostasis is critical for preventing infections, inflammatory diseases, and tumor development. Recent findings in animal models of ulcerative colitis (UC) have revealed key mechanisms by which *Sarcandra glabra* regulates mucosal immunity. Studies have shown that *S. glabra* extract promotes intestinal epithelial cell repair and upregulates the expression of tight junction proteins claudin−1 and occludin, thereby restoring and maintaining the physical integrity of the intestinal mucosa (101).

*S. glabra* extracts have been reported to reshape immune balance by modulating key immune cell subsets, specifically by regulating the balance between pro−inflammatory Th17 cells and regulatory T cells (Tregs) in mesenteric lymph nodes. This is achieved by inhibiting the differentiation of pathogenic T helper 17 (Th17) cells and the production of the related cytokine interleukin−17 (IL−17), while promoting the differentiation of immunosuppressive regulatory T cells (Tregs). This process is closely associated with the regulation of the IL−17/Notch1/FoxP3 signaling pathway. Preliminary studies also indicate that *S. glabra* enhances the phagocytic function of macrophages and may modulate innate immunity through signaling pathways such as NF−κB. However, the vast majority of current studies are still limited to animal models, and high−quality human clinical trial data remain scarce (102).

In an *in vitro* study using human osteosarcoma MG−63 cells, Zhang et al. (103) demonstrated that the acidic polysaccharide SGP−2 from *S. glabra* can directly inhibit the malignant biological behavior of tumor cells. Another study included a blank control group, gradient concentration SGP−2 treatment groups (62.5–250 nM), and a positive control group (doxorubicin). Scratch wound healing assay results showed that within the concentration range of 31.25–125 nM, the wound healing area decreased significantly with increasing concentration, with the most pronounced migration inhibition observed at 125 nM. The molecular mechanism involves downregulation of the receptor for advanced glycation end products (RAGE) and inhibition of the NF−κB signaling pathway. At 125 nM, SGP−2 reduced RAGE expression by 66.2%, while also inhibiting nuclear translocation of NF−κB p65 and phosphorylation of IKKα/β, thereby blocking the transduction of tumor cell migration signals. These findings suggest that *S. glabra* polysaccharides may regulate innate immunity through the NF−κB signaling pathway (101). However, the vast majority of current studies are still limited to animal models, and high−quality human clinical trial data remain scarce.

#### 4.6.2 Other immunomodulatory effects

The immune system plays a critical role in host defense against tumors, and many natural products exert anticancer effects by modulating immune responses (104). Numerous studies have demonstrated that *S. glabra* has immunomodulatory properties that enhance both innate and adaptive immunity in a dose-dependent manner. Within the concentration range of 6.25 to 100 mg/L, Zhongjiefeng injection significantly inhibited the proliferation of Hep−A−22 cells after 48 h of treatment in a concentration−dependent manner, with an IC_50_ of 56 mg/L. Different doses of Zhongjiefeng exerted different effects on immune function; at a dose of 10 mg/10 g¹, it significantly increased immune organ indices. All Zhongjiefeng treatment groups at various concentrations increased peripheral white blood cell counts without significantly reducing body weight, a prominent advantage not possessed by conventional cytotoxic drugs. These findings indicate that Zhongjiefeng injection has low toxicity, providing a theoretical basis for its clinical application (105).

Animals were randomly divided into a normal control group, a high−dose Zhongjiefeng tablet group (3.6 g/kg), and a low−dose Zhongjiefeng tablet group (1.8 g/kg), to evaluate the effect of Zhongjiefeng tablets on the phagocytic function of the reticuloendothelial system in mice. The results showed that the high−dose Zhongjiefeng tablet group significantly increased the carbon clearance index compared with the normal control group (P < 0.05). These findings indicate that Zhongjiefeng tablets can enhance the phagocytic function of the non−specific immune reticuloendothelial system in mice, which may be beneficial for eliminating or inhibiting tumor cells (106).

Clinical observations suggest that daily intravenous administration of Zhongjiefeng injection for 15 days consecutive days alleviates cancer-related pain, likely via immune enhancement (107).

Administration of *Sarcandra glabra* extract (125, 500 mg/kg/d) orally to restrained mice for five consecutive days alleviated stress−induced reductions in lymphocyte count, the balance of CD4^+^ T/CD8^+^ T cells, and splenic NK cell activity. SGE also significantly reduced the level of lipid peroxidation and increased the oxygen radical absorbance capacity (ORAC) in splenocytes. These results indicate that SGE extract modulates the stress−weakened immune response by enhancing the antioxidant capacity of immune cells, suggesting dual immunomodulatory and antioxidant effects (108).

The total flavonoid glycosides in *S. glabra* also exhibit regulatory effects on immune function similar to those of ginseng. At low doses, they boost immunity in immunocompromised individuals, while at high doses, they help attenuate excessive immune activation, thus maintaining homeostasis without toxicity or side effects such as microbial imbalance or organ dysfunction.

**Figure 2:**
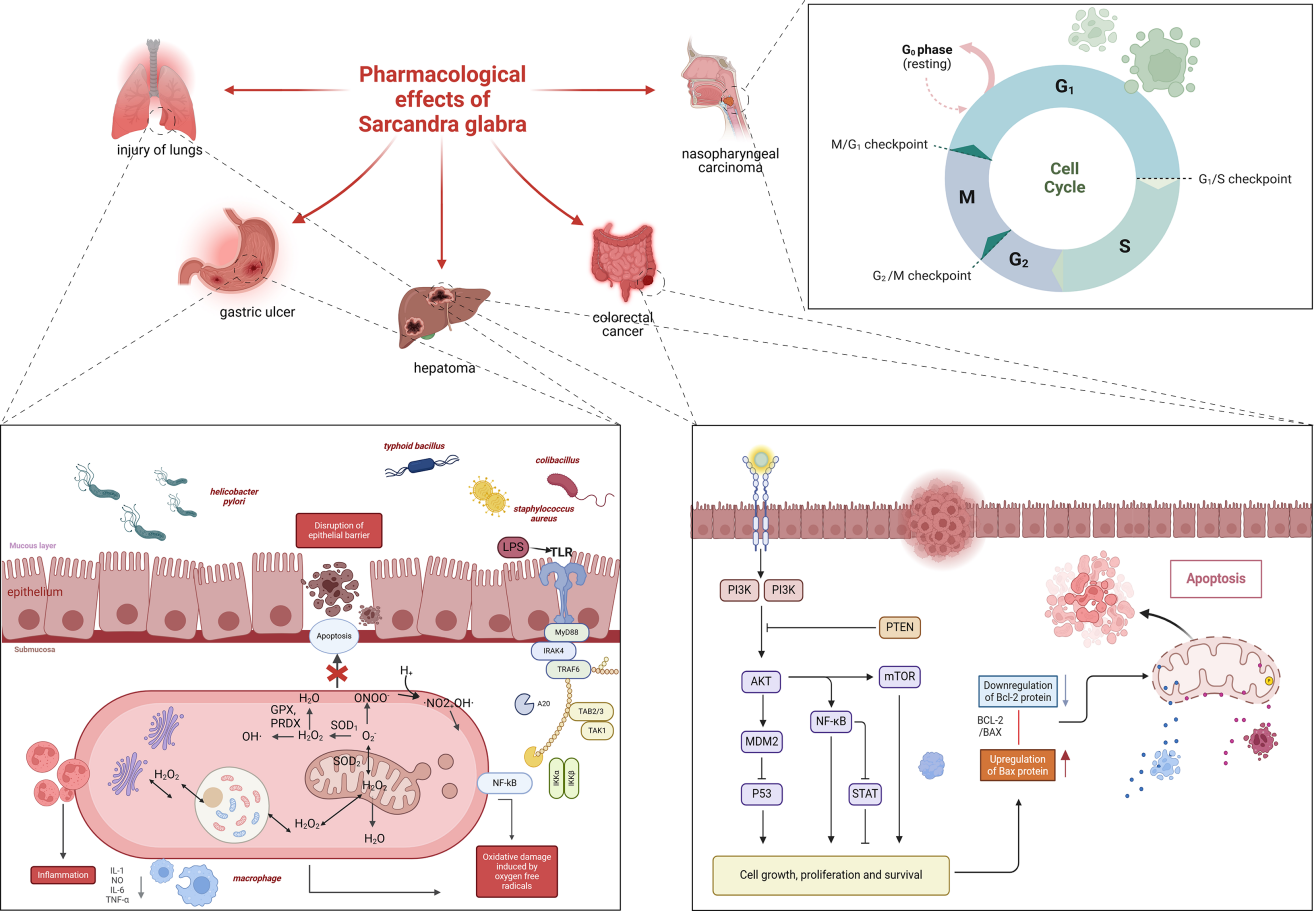
Mechanisms underlying the pharmacological activities of Sarcandra glabra in digestive system diseases. Sarcandra glabra exerts multi-target therapeutic effects across several digestive system diseases, including gastric ulcer, hepatoma, colorectal cancer, and nasopharyngeal carcinoma. Its gastroprotective actions involve attenuation of pathogen-induced epithelial barrier disruption, suppression of TLR/NF-κB-mediated inflammatory signaling, and enhancement of intracellular antioxidant defenses against ROS accumulation. Its antitumor effects are mediated through coordinated modulation of the PI3K/AKT/mTOR and NF-κB/STAT pathways, P53 stabilization, induction of Bcl-2/Bax-dependent apoptosis, and cell cycle arrest.

Polysaccharide extracts from *S. glabra* enhance the secretion of both pro- and anti-inflammatory cytokines by upregulating membrane protein-related immune molecules in RAW264.7 cells (56). The acidic polysaccharide fraction, in particular, activates dendritic cells via TLR4 signaling, thereby enhancing humoral and cellular immune responses (57). This is consistent with the established role of TLR4 as a key pattern recognition receptor that bridges innate and adaptive immunity; TLR4 agonists have been successfully used as vaccine adjuvants to promote dendritic cell maturation and T cell activation (109). The acidic polysaccharide, in particular, activates dendritic cells via TLR4 signaling, thereby enhancing humoral and cellular immune responses. These findings highlight the potential of *S. glabra* as an immunoadjuvant in cancer immunotherapy. The pharmacological effects of various Zhongjiefeng preparations and their active ingredients are summarized in **Table 5**. As summarized in **Figure 2**, the pharmacological effects of *S. glabra* are mediated through multiple interconnected pathways. The anti−inflammatory effect is mainly attributed to suppression of the LPS/TLR4/NF−κB axis, leading to reduced production of IL−1, IL−6, TNF−α, and NO. Meanwhile, the anti−tumor effect involves regulation of the PI3K/AKT/p53 pathway, shifting the Bcl−2/Bax balance toward apoptosis and causing cell cycle arrest at the G_1_/S checkpoint. The integrated view in **Figure 2** highlights the multi−target nature of *S. glabra* in treating digestive system diseases.

**Table 5:** Pharmacological effects of Zhongjiefeng preparations and their active ingredient.

Name	Pharmacological effect	Mechanism of action	Literature
Rosmarinic acid and astilbin	Anti-ulcer effect	Gastroprotective	(55)
Herba Pileae Scriptae polysaccharide extract	Immunomodulation	Immunomodulation, Cytokine-membrane synergy	(56)
Sarcandra glabra acidic polysaccharide	Immunomodulation	TLR4-immunopotentiation, Vaccine Immunopotentiation	(57)
Sarcandra glabra extract	Antibacterial effect, Anti-ulcer effect	Barrier fortification, Membrane stabilization, Gastroprotective	(65, 82)
Zhongjiefeng injection	Anti-inflammatory Action, Anti-ulcer effect, Anti-gastric cancer effect, Anti-liver cancer effect, Anti-colorectal cancer effect, Immunomodulation	IL-1 inhibition, Inflammation blocking, Antiviral, Antibacterial, Restorative chemoprevention, Gastroreparative activity, Mucosal regeneration, Apoptosis induction, Cytostatic immunomodulation, Cytostatic and Apoptotic Effects, Bax-mediated apoptosis, Cytostatic and Apoptotic Effects, Enhances immunity, Cancer pain alleviation	(81, 91, 94, 97, 107, 110–112)
Herba Sarcandrae Alcohol extract	Anti-nasopharyngeal carcinoma effect	Cytostatic and Apoptotic Effects, Antiproliferative effect	(85, 86)
Total flavonoids	Anti-gastric cancer Activity	Chemopreventive defense, Immunochemoprevention, Onco-gastroprotection	(95)
Caffeic acid-3,4-dihydroxybenzen	Anti-liver cancer effect	GSK-3β reprogramming, Proteostatic suppression	(98)
Herba Sarcandrae Chinese medicinal preparation	Anti-inflammatory Action	IL-1 inhibition, Inflammation blocking, Antiviral, Antibacterial	(110)
Isofraxidin	Anti-colorectal cancer effect	Blocks Akt signaling, Antiproliferative Effect	(113)
Total flavonoid glycosides	Immunomodulation	Enhances phagocytosis (ginseng-like effect),dose-dependently modulates immune function	(114)

## 5 Clinical application of *Sarcandra glabra* in digestive system diseases

The clinical evidence for *S. glabra* in digestive system diseases varies widely in quality. In this section, we prioritize findings from high−quality randomized controlled trials (RCTs) where available. *Sarcandra glabra* has been clinically used for the treatment of digestive system diseases such as acute and chronic dysentery, bacterial infectious diarrhea, rotavirus enteritis in children, acute gastroenteritis, duodenal ulcer infected by Helicobacter pylori and gastric ulcer, and has achieved satisfactory results (115). Clinical practice has proved (116) that Zhongjiefeng injection also shows good efficacy in the treatment of common inflammatory diseases, such as appendicitis, bacillary dysentery, acute gastroenteritis, abscess, etc., and also shows significant effect in the prevention of postoperative infection.

### 5.1 Treatment of infectious diarrhea

#### 5.1.1 Bacterial enteritis and bacillary dysentery

Bacillary dysentery, primarily caused by *Shigella species*, remains a significant public health concern, especially with the increasing resistance to conventional antibiotics. Clinical studies have demonstrated that *S. glabra* and its preparations, such as Caoshanhu Tablets and Xiaoyanling Tablets, exhibit potent antibacterial activity against *Shigella flexneri* and *Shigella sonnei*. Notably, Crystal A, the active ingredient isolated from *S. glabra*, has shown both anti-inflammatory and antibacterial effects, sensitive to *Shigella flexneri* and *Shigella sonnei* (117). In a small uncontrolled clinical study (n=33), Caoshanhu Tablets achieved complete remission in all acute cases and improvement in chronic cases, with no reported adverse effects. However, due to the lack of a control group and randomization, these findings should be interpreted as preliminary, and further high−quality RCTs are needed to confirm the safety and efficacy of this preparation.

#### 5.1.2 Viral enteritis and rotavirus-induced infantile diarrhea

Rotavirus (RV) is the leading cause of viral enteritis in infants and young children. It disrupts intestinal epithelial integrity and contributes to microbiota imbalance (118–120). Clinical studies have shown that intravenous administration of *S. glabra* significantly alleviates symptoms by promoting mucosal regeneration, restoring gut flora, and improving water and electrolyte absorption. One randomized controlled trial (RCT) reported that the combination of Zhongjiefeng injection with Biqi achieved a significantly higher efficacy rate (96.1%) compared to ribavirin alone (71.3%) (121, 122). Although this suggests a potential benefit, independent validation in larger, well−designed RCTs is needed. Similar positive outcomes were reported when *S. glabra* was combined with anisodamine or Smecta, with no significant adverse reactions (123, 124).

#### 5.1.3 Intestinal flora modulation and mucosal repair

Beyond its direct antiviral and antibacterial properties, *S. glabra* may help restore intestinal homeostasis by modulating the gut microbiota and enhancing mucosal barrier integrity. In inflammatory bowel diseases (IBD), the gut microbiota shows reduced diversity, depletion of butyrate-producing bacteria, and expansion of pathobionts. These changes collectively disrupt mucosal barrier function (125). Preliminary clinical observations suggest that *S. glabra* may promote the reestablishment of microbial diversity and mucosal integrity, potentially contributing to symptom resolution and reduced disease recurrence (126, 127). However, these findings are based on small descriptive studies, and no high−level evidence currently supports this claim. Further research is needed before drawing firm conclusions.

### 5.2 Treatment of gastrointestinal immune-inflammatory disorders

#### 5.2.1 Infantile mesenteric lymphadenitis

A small observational study reported that adding Zhongjiefeng injection to conventional treatment was associated with improved outcomes in children with mesenteric lymphadenitis. The authors attributed this to the antibacterial, antiviral, and immunoregulatory properties of *S. glabra*. However, due to the lack of randomization and blinding, these results are preliminary and require confirmation in well−controlled trials. It enhances pathogen clearance and supports lymphoid immune responses without significant adverse effects (128).

#### 5.2.2 Acute and chronic pharyngolaryngitis

Compound *S. glabra*-based atomized formulations have demonstrated efficacy in both acute and chronic pharyngitis. In randomized controlled studies, patients treated with aerosolized *S. glabra* experienced more rapid symptom relief compared to conventional treatments. These benefits are attributed to its anti-inflammatory and mucosal reparative effects.

A randomized but non−blinded study by Liao et al. (129) assigned 90 patients with wind−heat type acute pharyngitis to receive either aerosolized compound *S. glabra* plus acupoint injection or a control treatment. The treatment group showed better clinical outcomes with minimal side effects. Nevertheless, the lack of blinding and small sample size limit the strength of this evidence. Similarly, Li Yunsong et al. evaluated 80 patients with chronic pharyngitis and found those treated with *S. glabra* atomization (5 mL of compound solution and 20 mL of 0.9% sodium chloride injection) exhibited greater improvement in clinical signs and symptoms compared to patients receiving Manyanshuling Qinghouliyan Granules. These results support the application of compound *S. glabra* aerosol as an effective and well-tolerated intervention for chronic pharyngitis (130).

#### 5.2.3 Gastritis and gastric ulcer

Gastric ulcer, a subtype of peptic ulcer, results from degradation of the gastric mucosa by hydrochloric acid and pepsin (131). Its causes are often associated with dietary habits, medical history, and *Helicobacter pylori* infection. While traditional treatment strategies primarily focus on inhibiting gastric acid secretion and protecting gastric mucosa (132), *S. glabra* offers a complementary mechanism of action through its analgesic, anti-inflammatory, and antimicrobial activities. Specifically, *S. glabra* demonstrates inhibitory effects against *H. pylori*, the primary pathogen implicated in ulcer pathogenesis. When used in conjunction with traditional anti-ulcer agents such as omeprazole, *S. glabra* alleviates ulcer-related pain and promotes mucosal healing. Omeprazole’s acid-suppressing effect may also facilitate optimal conditions for *S. glabra*-mediated tissue repair (133). In addition, the immunomodulatory and potential anti-tumor properties of *S. glabra* may enhance the host resistance and prevent the risk of malignant transformation in chronic ulcer cases.

Clinically, combination therapy involving *S. glabra*, Weifuchun and folic acid has shown promising outcomes in patients with chronic atrophic gastritis with positive for *H. pylori*. Eradication of the pathogen is considered essential for improving prognosis and preventing progression to gastric cancer. A non−randomized comparative study suggested that replacing metronidazole with *S. glabra* in triple therapy regimens (e.g., amoxicillin + bismuth potassium citrate + *S. glabra*) might reduce adverse effects and improve patient adherence. However, these findings are preliminary due to the lack of randomization and control for confounding factors. Additionally, Zhongjiefeng Granules have significant therapeutic efficacy in gastric ulcer management, especially when used alongside Western medicine, resulting in reduced recurrence rates and improved clinical outcomes (134).

### 5.3 Applications in digestive system tumors

In recent years, with the development of new technology of TCM preparation, Zhongjiefeng injection has been widely used in the field of anti-tumor treatment. Zhongjiefeng preparations have curative effects on pancreatic cancer, rectal cancer, liver cancer, esophageal cancer and other digestive system malignant tumors (135, 136). Among these, the clinical response has been particularly notable in pancreatic cancer and gastric cancer, followed by rectal cancer, liver cancer and esophageal cancer, and bladder cancer, gallbladder cancer and nasal tumors. A clinical summary (137) that pooled data from small, uncontrolled case series reported response rates of 70% for pancreatic cancer, 67.7% for gastric cancer, 59.1% for rectal cancer, 44% for liver cancer, and 40.9% for esophageal cancer. These figures should be interpreted with extreme caution, as they are not derived from randomized controlled trials and may be subject to selection bias. In addition to its direct antitumor effects, *S. glabra* injection can also be used in combination with chemotherapy drugs, which not only reduces the dosage of radiotherapy and chemotherapy drugs, but also reduces the toxic and side effects on the blood and immune system, with significant therapeutic effect (106).

The incidence of hematological toxicity and digestive tract reactions in the treatment group was significantly lower than that in the control group. Furthermore, hypersensitivity reactions such as skin itching, rashes and other allergic reactions were infrequent or absent. These results show that *S. glabra* injection can significantly improve the quality of life in patients with advanced-stage disease. By reducing adverse drug reactions and enhancing the overall therapeutic effect, *S. glabra* serves as a safe and effective component of integrative cancer therapy (138).

#### 5.3.1 Nasopharyngeal carcinoma

Multiple clinical studies have demonstrated that *S. glabra*, when used as an adjuvant to radiotherapy and chemotherapy, significantly reduces treatment-related toxicities in patients with locally advanced nasopharyngeal carcinoma. Several small observational studies have reported that adding *S. glabra* to chemoradiotherapy may reduce treatment−related toxicities in nasopharyngeal carcinoma patients. For example, Huang Dongning (139) and Huang Yibo (140) noted improvements in radiation−induced xerostomia and mucositis in small cohorts. However, these studies lacked control groups and randomization, and therefore provide only low−level evidence. Well−designed RCTs are needed to confirm these benefits. Tang Chunyuan’s investigation highlighted the antioxidative effects of *S. glabra* extract in patients with stage III–IVa nasopharyngeal carcinoma undergoing chemoradiotherapy, showing satisfactory tumor regression and attenuation of treatment-induced toxicity (141). Ma Shanshan further confirmed that *S. glabra* could reduce parotid gland injury, alleviate both acute and chronic xerostomia, and prevent radiation-induced dental caries, all without negatively affecting survival outcomes (88). Additionally, Qin Yutao et al. found that *S. glabra* effectively alleviated 5-fluorouracil-induced bone marrow suppression and oral mucositis, reinforcing its role as a cytoprotective agent during combination chemotherapy (142).

#### 5.3.2 Esophageal cancer

The clinical efficacy of Zhongjiefeng injection in advanced esophageal cancer has also been well-documented. One small non−randomized comparative study reported that the addition of Zhongjiefeng injection to chemotherapy increased the overall response rate from 70% to 93.55% and the disease control rate from 60% to 87.1%. Given the lack of randomization and potential confounding, these results should be considered exploratory. Importantly, patient-reported quality of life did not decrease following treatment, highlighting the formulations safety and tolerability. Mechanistic analysis found that Zhongjiefeng injection may suppress the expression of angiogenesis- and metastasis- related markers such as VEGF and S100A4, thereby exerting antitumor effects. However, while the addition of *S. glabra* improved efficacy, its impact on reducing chemotherapy-associated adverse events, such as nausea, hematologic toxicity, and organ dysfunction, was less pronounced (143).

#### 5.3.3 Gastric cancer

A small randomized trial (n=46) reported that combination therapy with Zhongjiefeng injection and lapatinib mesylate tablets produced superior outcomes compared to apatinib monotherapy. However, the limited sample size and lack of blinding reduce the confidence in these findings. Larger, well−designed RCTs are required (144). The clinical observation of Zhongjiefeng injection combined with docetaxel, cisplatin and 5-Fu in the treatment of advanced gastric cancer showed that the objective effective rate of Zhongjiefeng injection combined with chemotherapy was 62.1%, which was significantly improved compared with 31.0% in the control group (81). Additionally, the quality of life improvement rate increased from 45.3% to 75.9%, and patients demonstrated greater treatment tolerance and prolong survival. Guo Yuefeng’s research has further confirmed the value of Zhongjiefeng Injection in the treatment of gastric cancer. Among 32 patients with advanced gastric cancer, those treated with Zhongjiefeng injection plus chemotherapy showed significantly better physical recovery and weight gain compared to those receiving chemotherapy alone (83). Collectively, these results support the tumor-inhibiting, immune-enhancing, and chemotherapy-sparing roles of *S. glabra* in gastric cancer management.

#### 5.3.4 Colorectal cancer

A small uncontrolled study (n=35) suggested that Zhongjiefeng tablets may improve cellular immune function in postoperative colorectal cancer patients receiving chemotherapy (145). However, due to the lack of randomization, this evidence is preliminary and requires confirmation in well−controlled trials. At present, the research on the treatment of colorectal cancer by *S. glabra* mainly focuses on the discussion of pathological mechanism. Including the validation of their activities such as anti-proliferation, induction of apoptosis, inhibition of migration and invasion, and the study of relevant signaling pathways *in vitro* models. In contrast, clinical-stage studies are still limited, and existing reports are mainly limited to small-scale preliminary observations or case studies. The available preclinical data suggest the potential therapeutic value of *S. glabra* or its active ingredient in the treatment of colon cancer. However, more systematic and large-sample clinical studies are still needed to confirm its clinical efficacy, safety, optimal dosage regimen, and clarify its exact mechanism of action in human body. Future research should focus on bridging the gap between basic research and clinical transformation, and provide a more solid basis for the application of *S. glabra* in the clinical treatment of colon cancer.

## 6 Discussion and outlook

### 6.1 Summary of pharmacological and clinical efficacy

*Sarcandra glabra* is rich in bioactive constituents, including sesquiterpenes, coumarins, flavonoids, organic acids, polysaccharides and other active ingredients, which endows *S. glabra* with various pharmacological property, such as anti-inflammatory, antimicrobial, hemostatic, and immunomodulatory effects. These pharmacological advantages have enabled its clinical application in various digestive system diseases, including gastroenteritis, peptic ulcers, bacillary dysentery, and intestinal infections. Multiple formulations (e.g., injections, tablets, granules, oral liquids) derived from *S. glabra* have demonstrated therapeutic effectiveness and convenience in clinical settings. Moreover, its use in adjuvant cancer therapy, especially for gastrointestinal tumors such as nasopharyngeal carcinoma, gastric cancer, liver cancer, and colorectal cancer, has further broadened its therapeutic spectrum.

### 6.2 Strengths and opportunities

One of the key advantages of *S. glabra* lies in its multi-target, multi-pathway mechanism, which enables comprehensive symptom control while maintaining a favorable safety profile. Compared with conventional western therapies, especially antibiotics and cytotoxic drugs, *S. glabra* exhibits fewer side effects and better patient compliance. Notably, it has shown efficacy against antibiotic-resistant pathogens and viral agents, presenting a valuable option for managing infectious and inflammatory gastrointestinal conditions. Furthermore, its anti-ulcer, immunomodulatory, and anticancer activities highlight its potential in integrated digestive disease management.

### 6.3 Safety and toxicology

Although *S. glabra* has been widely used clinically, systematic safety evaluation remains incomplete, with long−term data particularly scarce. *S. glabra* is generally well tolerated in short−term use. Reported mild events include nausea, mild gastrointestinal discomfort. No hepatotoxicity or nephrotoxicity has been convincingly attributed to *S. glabra*.

A toxicological study by Sun et al. (146) demonstrated that the oral LD_50_ of *S. glabra* in mice was within the non−toxic range. Furthermore, the mouse sperm abnormality test, bone marrow micronucleus test, and Ames test all yielded negative results, suggesting no mutagenic potential. Based on TCM theory and clinical experience, *S. glabra* should be avoided during pregnancy and in patients with known hypersensitivity to plants of the Chloranthaceae family.

Despite these findings, published data on adverse reactions, toxicity, and contraindications of *S. glabra* remain limited. Therefore, future pharmacovigilance and well−designed toxicological investigations are urgently needed to better characterize its safety profile and guide rational clinical use.

### 6.4 Current challenges and limitations

At present, the research and application of *S. glabra* in the treatment of leukemia and other fields have been relatively in-depth, but the research on digestive system diseases is relatively small. Although *S. glabra* and its compound preparations have shown reported effects in the prevention and treatment of digestive system diseases in limited clinical practice, the number of related clinical trials is small and the sample size is relatively limited. This limits the full assessment of its efficacy and safety. Although the safety of short-term use of *S. glabra* is high, there is a lack of safety data for long-term use, especially in large-scale populations. On the other hand, the efficacy of *S. glabra* is the result of multi-component, multi-pathway and multi-target synergy, which contains many known and unknown components. The chemical composition of *S. glabra* is complex, and different producing areas and different processing methods may lead to differences in composition and content, which may affect the consistency and reliability of its clinical application. Although it has been shown that *S. glabra* has a variety of pharmacological effects, such as anti-inflammatory, antibacterial and anti-tumor, its specific molecular mechanism has not been fully clarified. Ferroptosis is a key regulator of tumor progression, immune microenvironment, and treatment response in digestive tract malignancies (72). Therefore, future studies should investigate whether *S. glabra* or its active components exert anti-tumor effects by inducing or modulating ferroptosis. Further mechanistic studies will help to better understand its efficacy and safety. *S. glabra* is often used in combination with other drugs, but studies on herb-drug interactions are limited. This lack of data means that potential risks cannot be ruled out, and safety monitoring is warranted. In general, the mechanism of active components, structure-activity relationship, metabolic process *in vivo*, targets and pathways of *S. glabra* has not been studied in depth, which limits its scientific application in the prevention and treatment of digestive system diseases to a certain extent.

Intestinal flora imbalance is closely related to many digestive system diseases, such as irritable bowel syndrome (IBS), intestinal infection, colorectal cancer and so on. The composition and function of intestinal flora can significantly affect the efficacy of TCM. Studies haves shown that Pien Tze Huang, a traditional medicine, suppresses Colorectal Tumorigenesis through Restoring Gut Microbiota and Metabolites. TCM can exert its beneficial function by regulating intestinal flora and its metabolites, and intestinal flora can also be transformed into bioactive metabolites by TCM ingredients, thus enhancing the therapeutic effect of TCM. *S. glabra* contains antibacterial and anti-inflammatory compounds that may promote repair of the digestive tract mucosa and ulcer healing. In addition, its immunomodulatory properties could potentially help prevent intestinal infections, inflammatory diseases, and possibly tumor development, though direct evidence is still limited. *S. glabra* may exert its effects by inhibiting harmful bacteria, restoring gut microbiota balance, and enhancing systemic immune responses. A better understanding of the bidirectional interaction between *S. glabra* and the gut microbiota is therefore important for explaining its pharmacological properties in digestive diseases.

### 6.5 Future research directions

In order to further explore the effect and mechanism of *S. glabra* on digestive system diseases, we need to systematically study *S. glabra* and its compound preparations from multiple perspectives, levels and ranges. These include the mechanistic elucidation of its bioactive constituents and their specific molecular targets, as well as the standardization and quality control of its formulations across different sources and manufacturing processes. In addition, large-scale, multicenter randomized controlled trials are essential to rigorously assess its efficacy and safety across diverse patient populations. Pharmacokinetic and pharmacodynamic studies, including the evaluation of potential herb-drug interactions, are also urgently needed to support safe integration into clinical practice. Moreover, the application of systems biology and multi-omics technologies-such as metabolomics and gut microbiome profiling-will be instrumental in uncovering how *S. glabra* modulates key physiological pathways, particularly within the gut-liver and gut-tumor axes. It is necessary to further study its mechanism of action, optimize its dosage form and clarify its indications, so as to screen out more valuable active ingredients and further develop and utilize its medicinal value. In addition, *S. glabra* also has the potential to improve the interaction of intestinal flora, which is worth further exploration.50.

### 6.6 Conclusion

In summary, this review systematically summarizes the active constituents of *S. glabra* and their pharmacological activities and clinical applications in digestive system diseases. To date, over 100 compounds have been identified in *S. glabra*, including sesquiterpenes, coumarins, flavonoids, organic acids, and polysaccharides, providing a chemical basis for its bioactivities. *S. glabra* exerts multi−target effects against digestive system diseases through several complementary mechanisms. It possesses direct antibacterial activity against *Helicobacter pylori*, Shigella, and Staphylococcus aureus. Its anti−inflammatory action is mediated via the TLR4/NF−κB and MAPK signaling pathways. Gastric protection is achieved by promoting mucosal repair, upregulating tight junction proteins (claudin−1 and occludin), and exerting antioxidant activity (e.g., rosmarinic acid). Immunomodulation occurs through regulation of the Th17/Treg balance, enhancement of SIgA secretion, and activation of dendritic cells. Its anti−tumor effects are mediated by induction of apoptosis, cell cycle arrest, and inhibition of telomerase activity.

Current small−scale studies and a few randomized controlled trials suggest potential benefits of *S. glabra* in treating infectious diarrhea, gastritis, and peptic ulcers, as well as its use as an adjuvant therapy for nasopharyngeal, gastric, and colorectal cancers. However, most clinical data are derived from low−level evidence (e.g., small sample sizes). *S. glabra* is generally well tolerated in short−term use, with mild gastrointestinal discomfort being the most common adverse event. Toxicological studies indicate low acute toxicity and no mutagenicity. Nevertheless, data on long−term safety, chronic toxicity, and reproductive toxicity remain incomplete. High−quality, large−scale randomized controlled trials are urgently needed, along with systematic pharmacovigilance and standardized toxicological evaluations. Mechanistic studies should focus on gastrointestinal mucosal immunity and gut microbiota modulation.

In conclusion, *S. glabra* is a promising herbal medicine for digestive system diseases, with well−defined chemical diversity and multi−target mechanisms, particularly in regulating mucosal immunity and protecting the intestinal barrier. However, current evidence is preliminary, and rigorous clinical and toxicological studies are essential before it can be recommended for routine clinical use.

## References

[1] Leng Y Lv G Chen S . Research related to the effectiveness of anti-tumor effect of Herba Sarcandrae. Chin J Modern Drug Appl. (2010) 4:232–4. doi:10.14164/j.cnki.cn11-5581/r.2010.06.030

[2] Shao J Li Y Xie B Chen F Zhou L Liu W . UPLC-LTQ-Orbitrap-based determination of dynamic accumulation of chemical constituents in Sarcandra glabra. Chin Traditional Patent Med. (2021) 43:2114–9. doi:10.3969/j.issn.1001-1528.2021.08.025

[3] Tsui W Brown G . Cycloeudesmanolides from sarcandra glabra. Phytochemistry. (1996) 43:819–21. doi:10.1016/0031-9422(96)00352-4

[4] Hu X Yang J Xu X . Three novel sesquiterpene glycosides of Sarcandra Glabra. Chem Pharm Bull. (2009) 57:418–20. doi:10.1248/cpb.57.418 19336941

[5] Li X Shen J Jiang Y Shen T You L Sun X . Anti-inflammatory effects of Chloranthalactone B in LPS-stimulated RAW264.7 cells. Int J Mol Sci. (2016) 17:1938. doi:10.3390/ijms17111938 27879664 PMC5133933

[6] Tang P Zhao S Wang X Wang Y Kong L Luo J . Chloranthalactone B covalently binds to the NACHT domain of NLRP3 to attenuate NLRP3-driven inflammation. Biochem Pharmacol. (2024) 226:116360. doi:10.1016/j.bcp.2024.116360 38871334

[7] Zheng X Liu H Zhong H . Study on the chemical components of Coral Grass. Natural Product Res Dev. (2014) 26:1221–1224, 1284. doi:10.16333/j.1001-6880.2014.08.015

[8] Zhu L Li Y Yang J Zuo L Zhang D . Two new sesquiterpene lactones from Sarcandra glabra. J Asian Nat Prod Res. (2008) 10:541–5. doi:10.1080/10286020801966773 18470806

[9] Li Y Zhang D Li J Yu S Li Y Luo Y . Hepatoprotective sesquiterpene glycosides from Sarcandra glabra. J Nat Prod. (2006) 69:616–20. doi:10.1021/np050480d 16643038

[10] Colasurdo D Arancibia L Naspi M Laurella S . Using DP4+ probability for structure elucidation of sesquiterpenic lactones: The case of (-)‐Istanbulin A. J Phys Org Chem. (2022) 35:27–32. doi:10.1002/poc.4282 41531421

[11] Tian S Yu H . Atractylenolide II inhibits proliferation, motility and induces apoptosis in human gastric carcinoma cell lines HGC-27 and AGS. Molecules. (2017) 22:1886. doi:10.3390/molecules22111886 29099789 PMC6150195

[12] Bailly C . Atractylenolides, essential components of Atractylodes-based traditional herbal medicines: Antioxidant, anti-inflammatory and anticancer properties. Eur J Pharmacol. (2021) 891:173735. doi:10.1016/j.ejphar.2020.173735 33220271

[13] Deng M Chen H Long J . Atractylenolides (I, II, and III): a review of their pharmacology and pharmacokinetics. Arch Pharmacal Res. (2021) 44:633–54. doi:10.1007/s12272-021-01342-6 34269984

[14] Ramesh S Mehta G . A total synthesis of sarcandralactone A: a general, concise, RCM enabled approach to lindenanolide sesquiterpenoids. Tetrahedron Lett. (2015) 56:3941–4. doi:10.1016/j.tetlet.2015.04.132 38826717

[15] He X Yin S Ji Y Su Z Geng M Yue J . Sesquiterpenes and dimeric sesquiterpenoids from Sarcandra glabra. J Nat Prod. (2010) 73:45–50. doi:10.1021/np9006469 20038159

[16] Wang P Li R Liu R Jian K Yang M Yang L . Sarglaperoxides A and B, sesquiterpene-normonoterpene conjugates with a peroxide bridge from the seeds of Sarcandra glabra. Org Lett. (2016) 18:832–5. doi:10.1021/acs.orglett.6b00112 26824697

[17] Annunziata F Pinna C Dallavalle S Tamborini L Pinto A . An overview of coumarin as a versatile and readily accessible scaffold with broad-ranging biological activities. Int J Mol Sci. (2020) 21:4618. doi:10.3390/ijms21134618 32610556 PMC7370201

[18] Zhang L Xie Q Li X . Esculetin: A review of its pharmacology and pharmacokinetics. Phytotherapy Research: PTR. (2022) 36:279–98. doi:10.1002/ptr.7311 34808701

[19] Hui Y Wang X Yu Z Fan X Cui B Zhao T . Scoparone as a therapeutic drug in liver diseases: Pharmacology, pharmacokinetics and molecular mechanisms of action. Pharmacol Res. (2020) 160:105170. doi:10.1016/j.phrs.2020.105170 32877694

[20] Liu B Deng X Jiang Q Li G Zhang J Zhang N . Scoparone improves hepatic inflammation and autophagy in mice with nonalcoholic steatohepatitis by regulating the ROS/P38/Nrf2 axis and PI3K/AKT/mTOR pathway in macrophages. Biomedicine Pharmacotherapy. (2020) 125:109895. doi:10.1016/j.biopha.2020.109895 32000066

[21] Wang C Lu J Chen D Wang J Che K Zhong M . Comprehensive chemical study on different organs of cultivated and wild Sarcandra glabra using ultra-high performance liquid chromatography time-of-flight mass spectrometry (UHPLC-TOF-MS). Chin J Natural Medicines. (2021) 19:391–400. doi:10.1016/S1875-5364(21)60038-9 33941344

[22] Xu X Hu X Yuan J Yang J . Studies on chemical constituents of Sarcandra glabra. Zhongguo Zhong Yao Za Zhi. (2008) 33:900–2. doi:10.1080/10286020.2014.935348 18619347

[23] Majnooni M Fakhri S Shokoohinia Y Mojarrab M Afrakoti S Farzaei M . Isofraxidin: Synthesis, biosynthesis, isolation, pharmacokinetic and pharmacological properties. Molecules. (2020) 25:2040. doi:10.3390/molecules25092040 32349420 PMC7248759

[24] Yuan K Zhu J Si J Cai H Ding X Pan Y . Studies on chemical constituents and antibacterial activity from n-butanol extract of Sarcandra glabra. China J Chin Materia Med. (2008) 33:1843–6. 19007012

[25] Chen X Ying X Sun W Zhu H Jiang X Chen B . The therapeutic effect of fraxetin on ethanol-induced hepatic fibrosis by enhancing ethanol metabolism, inhibiting oxidative stress and modulating inflammatory mediators in rats. Int Immunopharmacol. (2018) 56:98–104. doi:10.1016/j.intimp.2018.01.027 29414667

[26] Chang B Jung Y Yoon C On J Hong J Kim Y . Fraxin prevents chemically induced hepatotoxicity by reducing oxidative stress. Molecules. (2017) 22:587. doi:10.3390/molecules22040587 28383514 PMC6154468

[27] Gao X Li X Zhang C Bai C . Scopoletin: a review of its pharmacology, pharmacokinetics, and toxicity. Front Pharmacol. (2024) 15:1268464. doi:10.3389/fphar.2024.1268464 38464713 PMC10923241

[28] Yang C Zhou Q Wu S . Scopolin obtained from Smilax China L. against hepatocellular carcinoma by inhibiting glycolysis: A network pharmacology and experimental study. J Ethnopharmacol. (2022) 296:115469. doi:10.1016/j.jep.2022.115469 35718053

[29] Wang Y Yan W Chen Q Huang W Yang Z Li X . Inhibition viral RNP and anti-inflammatory activity of coumarins against influenza virus. Biomedicine Pharmacotherapy. (2017) 87:583–8. doi:10.1016/j.biopha.2016.12.117 28081470

[30] Wu Z Zhang D Yuan Q Liu F Zhang H Shang G . Predictive analysis on quality markers for Sarcandrae Herba based on chemical constituents, pharmacological effects and network pharmacology. Modern Chin Med. (2023) 25:1113–24. doi:10.13313/j.issn.1673-4890.20220913002

[31] Liu L Mu Q Li W Xing W Zhang H Fan T . Isofraxidin protects mice from LPS challenge by inhibiting pro-inflammatory cytokines and alleviating histopathological changes. Immunobiology. (2015) 220:406–13. doi:10.1016/j.imbio.2014.10.007 25454811

[32] Li Y Yao J Han C Yang J Chaudhry T Wang S . Quercetin, inflammation and immunity. Nutrients. (2016) 8:167. doi:10.3390/nu8030167 26999194 PMC4808895

[33] Sohn U Lee S Lee H Nam Y Hwang W . The protective mechanism of quercetin-3-O-β-d-glucuronopyranoside (QGC) in H2O2–induced injury of feline esophageal epithelial cells. Arch Pharmacal Res. (2016) 39:1324–34. doi:10.1007/s12272-016-0808-7 27522656

[34] Takara K Kuniyoshi A Wada K Kinjyo K Iwasaki H . Antioxidative flavan-3-ol glycosides from stems of Rhizophora stylosa. Biosci Biotechnol Biochem. (2008) 72:2191–4. doi:10.1271/bbb.80065 18685199

[35] Mendes R Almeida S Soares I Barboza C Freitas R Brown A . Evaluation of the antioxidant potential of myricetin 3-O-α-L-rhamnopyranoside and myricetin 4'-O-α-L-rhamnopyranoside through a computational study. J Mol Model. (2019) 25:89. doi:10.1007/s00894-019-3959-x 30847605

[36] Alsufiani H Aldhaheri G Omar U Bahdilah T Mansouri R . Antioxidant activity and inhibitory effect of 2,4,4'-trihydroxychalcone on digestive enzymes related to obesity. Slovenian Veterinary Res. (2023) 60. doi:10.26873/SVR-1625-2022

[37] Bae S Lee J Hyun C . Anti-melanogenic and anti-inflammatory effects of 2'-hydroxy-4',6'-dimethoxychalcone in B16F10 and RAW264.7 cells. Curr Issues Mol Biol. (2024) 46:6018–40. doi:10.3390/cimb46060359 38921030 PMC11202956

[38] Kamisah Y Jalil J Yunos N Zainalabidin S . Cardioprotective properties of kaempferol: A review. Plants. (2023) 12:2096. doi:10.3390/plants12112096 37299076 PMC10255479

[39] Khajuria V Gupta S Sharma N Tiwari H Bhardwaj S Dutt P . Kaempferol-3-o-β-d-glucuronate exhibit potential anti-inflammatory effect in LPS stimulated RAW 264.7 cells and mice model. Int Immunopharmacol. (2018). 57:62–71. doi:10.1016/j.intimp.2018.01.041 29475097

[40] Xu W Li F Zhang X Wu C Wang Y Yao Y . The protective effects of neoastilbin on monosodium urate stimulated THP-1-derived macrophages and gouty arthritis in mice through NF-κB and NLRP3 inflammasome pathways. Molecules. (2022) 27:3477. doi:10.3390/molecules27113477 35684415 PMC9181946

[41] Athapaththu A Lee K Kavinda M Lee S Kang S Lee M . Pinostrobin ameliorates lipopolysaccharide (LPS)-induced inflammation and endotoxemia by inhibiting LPS binding to the TLR4/MD2 complex. Biomedicine Pharmacotherapy. (2022) 156:113874. doi:10.1016/j.biopha.2022.113874 36270256

[42] Xu Y . Study on the antioxidant effect of flavonoids extract from Coral Grass in Guizhou *in vitro*. South China Agric. (2017) 11:83–5. doi:10.19415/j.cnki.1673-890x.2017.31.024

[43] Gold R Linker R Stangel M . Fumaric acid and its esters: an emerging treatment for multiple sclerosis with antioxidative mechanism of action. Clin Immunol. (2012) 142:44–8. doi:10.1016/j.clim.2011.02.017 21414846

[44] Marchiosi R Ferro A Ramos A Baldoqui D Constantin R Constantin R . Calophyllum brasiliense Cambess: An alternative and promising source of shikimic acidCalophyllum brasiliense Cambess: Alternatives and promising sources of shikimic acid. Sustain Chem Pharm. (2019) 14:100188. doi:10.1016/j.scp.2019.100188 38826717

[45] Benali T Bakrim S Ghchime R Benkhaira N Omari N Balahbib A . Pharmacological insights into the multifaceted biological properties of quinic acid. Biotechnol Genet Eng Rev. (2022) 40:1–30. doi:10.1080/02648725.2022.2122303 36123811

[46] Ling X Zhang J Teng J Huang L Xia N . Effects of cross-linking of rice protein with ferulic acid on digestion and absorption of ferulic acid. Int J Food Sci Nutr. (2023) 74:313–26. doi:10.1080/09637486.2023.2199178 37076970

[47] Li X Zhang Y Yang L Feng Y Liu Y Zeng X . Studies of phenolic acid constituents from the whole plant of Sarcandra Glabra. Traditional Chin Drug Res Clin Pharmacol. (2012) 23:295–8. doi: 10.3969/j.issn.1003-9783.2012.03.015

[48] Tošović J Marković S Dimitrić Marković J Mojović M Milenković D . Antioxidative mechanisms in chlorogenic acid. Food Chem. (2017) 237:390–8. doi:10.1016/j.foodchem.2017.05.080 28764012

[49] Ehtiati S Alizadeh M Farhadi F Khalatbari K Ajiboye B Rahimi V . Promising influences of caffeic acid and caffeic acid phenethyl ester against natural and chemical toxins: A comprehensive and mechanistic review. J Funct Foods. (2023) 107:105637. doi:10.1016/j.jff.2023.105637 38826717

[50] Palomer X Delgado J Barroso E Carrera M . Palmitic and oleic acid: The yin and yang of fatty acids in type 2 diabetes mellitus. Trends Endocrinol Metab. (2018) 29:178–90. doi:10.1016/j.tem.2017.11.009 29290500

[51] Wei W Wong C Jia Z Liu C Ji F . Parabacteroides distasonis uses dietary inulin to suppress NASH via its metabolite pentadecanoic acid. Nat Microbiol. (2023) 8:1534–48. doi:10.1038/s41564-023-01418-7 37386075 PMC10390331

[52] Gu S Zheng H Xu Q Sun C Shi M Wang Z . Comparative toxicity of the plasticizer dibutyl phthalate to two freshwater algae. Aquat Toxicol. (2017) 191:122–30. doi:10.1016/j.aquatox.2017.08.007 28822891

[53] Zhang J Cui X Zhang M Bai B Yang Y Fan S . The antibacterial mechanism of perilla rosmarinic acid. Biotechnol Appl Biochem. (2022) 69:1757–64. doi:10.1002/bab.2248 34490944

[54] Kitamura N Yamamoto Y Yamamoto N Murase T . Rosmarinic acid ameliorates HCl-induced cystitis in rats. PloS One. (2023) 18:e0288813. doi:10.1371/journal.pone.0288813 37463180 PMC10353813

[55] Liu J Li X Lin J Li Y Wang T Jiang Q . Sarcandra glabra (Caoshanhu) protects mesenchymal stem cells from oxidative stress: a bioevaluation and mechanistic chemistry. BMC Complementary Altern Med. (2016) 16:423. doi:10.1186/s12906-016-1383-7 PMC5084467 27793132

[56] Jiang Z Chen Z Li X Zhao J Li S Hu J . Immunomodulatory effects of Sarcandra glabra polysaccharides on macrophage RAW264.7. Chin J Exp Traditional Med Formulae. (2014) 20:178–82. doi:10.13422/j.cnki.syfjx.2014120178

[57] Liu W Gong X Luo J Jiang L Lu W Pan C . A purified acidic polysaccharide from Sarcandra glabra as vaccine adjuvant to enhance anti-tumor effect of cancer vaccine. Carbohydr Polym. (2021) 263:117967. doi:10.1016/j.carbpol.2021.117967 33858570

[58] Jin L Guan X Liu W Zhang X Yan W Yao W . Characterization and antioxidant activity of a polysaccharide extracted from Sarcandra glabra. Carbohydr Polym. (2012) 90:524–32. doi:10.1016/j.carbpol.2012.05.074 24751073

[59] Huang R Xie P Shi J Yang J Ma C . Gas chromatography-mass spectrometry analysis of volatile oil from Sarcandra glabra. Chin Traditional Patent Med. (1998) 1:37–8. 37-38+53.

[60] Yang B Sun W Xue D Teng W . Determination of 12 elements in Sarcandra glabra by ICP-MS. Chin J Spectrosc Lab. (2011) 3:230–2. doi:10.1016/0165-1633(82)90087-9

[61] Mei Q Hu Y . Research progress on the pharmacological effects and clinical applications of Zhongjie Feng. Lishizhen Med Materia Med Res. (2011) 22:230–2. doi:10.4236/jbm.2022.108004

[62] Jiang W Kong X . Huang Renbin et al. Studies on anti-inflammatory and antibacterial effects of Tabellae Sarcandrae. J Guangxi Univ Chin Med. (2000) 1:50–2.

[63] Lu S . Research progress on Sarcandra glabra. J Chin Medicinal Materials. (2001) 8:606–8. doi:10.13863/j.issn1001-4454.2001.08.037

[64] Shao J . Study on the extraction, purification and pharmacological action of total flavonoids in Sarcandra glabra. In: Guizhou university Guizhou, China: Guizhou University (2008).

[65] Guo M . The efficacy of Sarcandra glabra extract alone or combined with antibiotics against helicobacter pylori *in vitro*. In: Nanchang university Nanchang, China: Nanchang University (2016).

[66] Wang A Ma X . Preliminary study on the active ingredients of Sarcandra glabra. Chin Traditional Herbal Drugs. (1979) 10:8–9. doi:10.15212/amm-2024-0050. 8-9+49.

[67] Wang J Du M . Comparison of external antibacterial effects of Sarcandra glabra tablets with Chaiyin, Shuanghuanglian, and Qutan oral liquids. Shanghai Med Pharm J. (2008) 2:80–2.

[68] Huang G . Clinical observation on the treatment of 42 cases of infant rotavirus enteritis with Zhongjiefeng. Hainan Med J. (2007) 8:111.

[69] Wen X . Clinical application progress of ZhongJieFeng. J Med Inf. (2005) 5:538–41.

[70] Li J Liang H Li X . The research progress of anti-infection effect of Sarcan draglabra. J Jiangxi Univ Chin Med. (2017) 29:113–6.

[71] Cheng H Li L Han D Shan F . The phytochemistry, pharmacology, and clinical, medicinal, and edible application of the genus Trollius: An updated systematic review. Food Med Homology. (2026). doi:10.26599/FMH.2027.9420142

[72] Guo J Wang Z Zhang Y Xie C Chen Y Ma L . The dual roles of ferroptosis in digestive tract tumors: Mechanisms, microenvironment regulation, and therapeutic integration with emphasis on immune interactions. Front Immunol. (2026) 17:1737847. doi:10.3389/fimmu.2026.1737847 41694363 PMC12901348

[73] Tsai Y Chen S Lin L Fu S . Anti-inflammatory principles from Sarcandra glabra. J Agric Food Chem. (2017) 65:6497–505. doi:10.1021/acs.jafc.6b05125 28110531

[74] Liu C Liu J Gu J Liu F Li J Yang B . Combination effect of three main constituents from Sarcandra glabra inhibits oxidative stress in the mice following acute lung injury: A role of MAPK-NF-κB pathway. Front Pharmacol. (2020) 11:580064. doi:10.3389/fphar.2020.580064 33597870 PMC7883675

[75] Sun S Yan Z Shui X Qi W Chen Y Xu X . Astilbin prevents osteoarthritis development through the TLR4/MD-2 pathway. J Cell Mol Med. (2020) 24:13104–14. doi:10.1111/jcmm.15915 33063931 PMC7701562

[76] Liu T Chen S . Sarcandra glabra combined with lycopene protect rats from lipopolysaccharide induced acute lung injury via reducing inflammatory response. Biomedicine Pharmacotherapy. (2016) 84:34–41. doi:10.1016/j.biopha.2016.09.009 27631138

[77] Wang M Zhao J Zhao Y Huang R Li G Zeng X . A new coumarin isolated from Sarcandra glabra as potential anti-inflammatory agent. Nat Prod Res. (2016) 30:1796–801. doi:10.1080/14786419.2015.1079186 26327462

[78] Zhu L Hong H . The effect of Zhongjie Feng on lipopolysaccharide induced IL-1 expression in endothelial cell lines. Zhejiang J Integrated Traditional Chin Western Med. (2007) 8:485–7.

[79] Wang H Yang M Zeng X Meng Z Han Q . Research progress in models of gastric mucosal lesion repair. Front Pharm Sci. (2022) 25:1431–5. doi:10.19962/j.cnki.issn1008-049X.2022.08.025

[80] Chen H Chen P Bao Y Ao J . 300 cases of protruding erosive gastritis treated with Zhujiefeng soft capsule. Chin J Integrated Traditional Western Med Digestion. (2012) 20:519–20. doi: 10.3969/j.issn.1671-038X.2012.11.015

[81] Li D Wang H . Observation on the adverse reactions and efficacy of Zhujiefeng injection combined with DCF regimen in the treatment of advanced gastric cancer. Inner Mongolia J Traditional Chin Med. (2013) 32:71–2. doi:10.16040/j.cnki.cn15-1101.2013.21.158

[82] Xu Z . Research overview of Coralleaf glabra. J Jiangxi Univ Chin Med. (1994) 1:37–7.

[83] Guo Y Huang L Wang S Jiao Z . 32 cases of intermediate and advanced gastric cancer treated with Zhujiefeng injection combined with chemotherapy. J Traditional Chin Med. (2003) 6:434. doi:10.13288/j.11-2166/r.2003.06.022

[84] Jiang L Li J . Study progress of anticancer effects of Sarcandrae Herba. Chin J Rational Drug Use. (2014) 11:29–31, 35. doi: 10.3969/j.issn.1672-5433.2014.04.008

[85] Kang M Tang A Liang G . A study on the inhibition of sarcandrae extracts on proliferation of nasopharyngeal carcinoma cell. J Guangxi Med Univ. (2008) 22:1132–7.

[86] Kang M Tang A Liang G . The inhibitory role of zhongjiefeng extracts on apoptosis and telomerase activity of nasopharyngeal carcinoma cell line exnograf in nude mice. J Clin Otorhinolaryngology Head Neck Surg. (2008) 3:347–9. doi:10.16190/j.cnki.45-1211/r.2008.03.053 19297858

[87] Qin J Wang R Teng J . The effect of Sarcandra glabra extracts on oxygen free radical. Lishizhen Med Materia Med Res. (2007) 12:2945–6. doi:10.1186/isrctn18094960 38164791

[88] Ma S Wang R Wei B Feng G Zhu X Li G . Curative effect observation of Sarcandra prevent poisonous side reaction induced by radiotherapy combined with neoadjunvant chemotherapy in nasopharyngeal carcinoma. Chin J Exp Traditional Med Formulae. (2010) 16:185–8. doi:10.13422/j.cnki.syfjx.2010.16.003

[89] Cui Y Kuang G Yue H Liu M Liang X . Experimental study on the inhibition of EBV capsid antigen expression in *in vitro* cells by Chinese herbal medicines. J Cancer Control Treat. (2001) 3:148–9.

[90] Zhao Y Sun Y Xiao B Chen Q . Antitumoral activity of Zhongjiefeng injection and its influence on the cell cycle of gastric cancer SGC-7901. Chin Traditional Patent Med. (2009) 31:997–1000.

[91] Zhao Y Sun Y Chen Q . Study on the inhibitory effect of Zhongjiefeng injection on human gastric cancer SGC-7901 transplanted tumors in nude mice and its induction of apoptosis. China Pharm. (2008) 20:8–9, 36.

[92] Wu J Lv S Lu C Gong J An J . Effect of 3,3′-biisofraxidin on apoptosis of human gastric cancer BGC-823 cells. Trop J Pharm Res. (2015) 14:1803–11. doi:10.4314/tjpr.v14i10.10

[93] Wu H Hu X Zhang X Chen S Yang J Xu X . Benzyl 2-β-glucopyranosyloxybenzoate, a new phenolic acid glycoside from Sarcandra glabra. Molecules. (2012) 17:5212–8. doi:10.3390/molecules17055212 22628042 PMC6268185

[94] He Z Zhong R Liu H . Effect of Zhongjiefeng injection combined with 5-FU on proliferation and apoptosis of gastric carcinoma cells MGC-803. Pract J Cancer. (2014) 29:1205–7. doi: 10.3969/j.issn.1001-5930.2014.10.002

[95] Zhang X Xin D . Clinical observation on the treatment of various stomach inflammations with Zhujiefeng tablets. Shanghai Med Pharm J. (2009), 424–5. doi: 10.3969/j.issn.1001-5930.2014.10.002

[96] Zhu X Long C Liang Y Peng Y Li T Non H . Observation on the effects of Sarcandrae compound on HepG2 cells. Guangxi Med J. (2012) 34:1597–9.

[97] Luo Y Wei Y Li T Chen X Wu L Wei Y . Effect of Zhuangjiefeng injection on apoptosis of human liver cancer cell line HepG2. Guangdong Med J. (2015) 36:1340–2. doi:10.13820/j.cnki.gdyx.2015.09.009

[98] Tang M Xie X Shi M Xin W Zheng G Zhang Y . Antileukemic effect of caffeic acid 3,4-dihydroxyphenetyl ester. Evidences for its mechanisms of action. Phytomedicine: Int J Phytotherapy Phytopharmacology. (2021) 80:153383. doi:10.1016/j.phymed.2020.153383 33091855

[99] Lv G Chen S Zhang Y Chen Y Lv X Hua B . Synergistic and attenuating effects of Sarcandra glabra volatile oil on chemotherapy in tumor-bearing mice. J Zhejiang Chin Med Univ. (2009) 33:116–8. doi:10.16466/j.issn1005-5509.2009.01.049

[100] Huang Y Zhao Y Yang Y Xiao B Chen Q . Anti-tumor effect of zhongjiefeng injection and its combination with adriamycin. Traditional Chin Drug Res Clin Pharmacol. (2007) 3:200–202, 216. doi:10.19378/j.issn.1003-9783.2007.03.010

[101] Zhang M Xu X Zhang Z Bi H Wang X Wang M . A review of Sarcandra Glabra (Thunb.) Nakai polysaccharides: Extraction, structural characteristics, pharmacology, and structure-activity relationships. J Ethnopharmacol. (2026) 359:121106. doi:10.1016/j.jep.2025.121106 41453545

[102] Zhong Z Deng P Luo X Zhu W Cui P Li Z . Multi-component characterization and quality evaluation strategy of sarcandrae herba by combining dual-column tandem hplc fingerprint and uplc-q-tof-ms/ms. Molecules. (2025) 30:1825. doi:10.3390/molecules30081825 40333856 PMC12029460

[103] Zhang Z Liu W Zheng Y Jin L Yao W Gao X . Sgp-2, an acidic polysaccharide from Sarcandra Glabra, inhibits proliferation and migration of human osteosarcoma cells. Food Funct. (2014) 5:167–75. doi:10.1039/c3fo60378d 24336744

[104] Ji Y Zhu X Wu J . The progress in the antitumor effect and mechanism of zhongjiefeng(Sarcandra Glabra). Guiding J Traditional Chin Med Pharm. (2016) 22:44–46, 55. doi:10.13862/j.cnki.cn43-1446/r.2016.09.015

[105] Sun W Li J Lan F Yang S . Antitumor effect of zhongjiefeng injection on mice liver cancer hep-a-22 and its toxicity. Chin Traditional Patent Med. (2003) 4:55–7.

[106] Jiang W Kong X Liang G Huang Z Chen J Huang R . Effects of tabellae sarcandrae on Malignant tumor and immunity. J Guangxi Med Univ. (2001) 1:39–41. doi:10.16190/j.cnki.45-1211/r.2001.01.017

[107] Yang H . 60 cases of cancer pain treated with zhongjiefeng injection. China Pharm. (2008) 20:59–60.

[108] He R Yao X Li H Dai Y Duan Y Li Y . The anti-stress effects of Sarcandra Glabra extract on restraint-evoked immunocompromise. Biol Pharm Bull. (2009) 32:247–52. doi:10.1248/bpb.32.247 19182384

[109] Crammond P Hastak P Delaney A Sasson S . Taking its toll: The role of toll-like receptor 4 in human health and disease, and its potential as a therapeutic target. Front Immunol. (2026) 17:1761361. doi:10.3389/fimmu.2026.1761361 41869302 PMC12999816

[110] Zhou B Liu K Chan J Chen C . Research progress on chemical constituents and pharmacological effects of the traditional chinese medicine Sarcandra glabra. Chin J Modern Appl Pharm. (2009) 26:982–6. doi:10.13748/j.cnki.issn1007-7693.2009.12.006

[111] Sun W Li J Lan F Yang S . Antitumor activity of zhongjiefeng injection on fc in mice with precarcinoma of stomach and its toxicity. Traditional Chin Drug Res Clin Pharmacol. (2003) 3:168–71. doi:10.19378/j.issn.1003-9783.2003.03.010

[112] Xie Y Yang G Qiu J Yan W . Experimental study of zhongjiefeng induced hct-8 colon cancer cell apoptosis. J Med Forum. (2018) 39:10–3, 16.

[113] Shen P Wang H Li M Ma Q Zhou C Pan F . Isofraxidin inhibited proliferation and induced apoptosis via blockage of akt pathway in human colorectal cancer cells. Biomedicine Pharmacotherapy. (2017) 92:78–85. doi:10.1016/j.biopha.2017.05.065 28531803

[114] Song L . Dictionary of modern chinese medicine. Beijing: People’s Health Publishing House (2001).

[115] Guo X Cao X . Pharmacological research and clinical application of sarcandra preparations. China Pharm. (2006) 1:73–5.

[116] The Research Leading Group of Swelling and Pain Disease of the Health Bureau of Guixi County, Jiangxi Province . A study on herbal sarcandra. Chin Traditional Herbal Drugs. (1972), 41–7.

[117] Fan X Bao Y Chen H Xu J Wang Q . Observation on the therapeutic effect of Sarcandra glabra tablets on 33 cases of bacterial dysentery. Chin Traditional Patent Med. (1983) 8:28. doi:10.3389/fmed.2024.1492108 39691367 PMC11649409

[118] Zhao G Wang R . Observation on the therapeutic effect of tongxin granules on 44 cases of infantile rotavirus enteritis. Chin Gen Pract. (2002) 9:675.

[119] Bidell M Hobbs A Lodise T . Gut microbiome health and dysbiosis: A clinical primer. Pharmacotherapy: J Hum Pharmacol Drug Ther. (2022) 42:849–57. doi:10.1002/phar.2731 36168753 PMC9827978

[120] Kuang W . Correlation analysis of neonatal rotavirus infection and gut microbiota. In: Xinjiang medical university. Xinjiang, China: Xinjiang Medical University. doi:10.1016/s0140-6736(82)92123-7

[121] Wang A Yuan X Bai J . Treatment of 53 cases of rotavirus enteritis in children with biqi and zhongjie feng. In: Journal of air force medical university Xi'an, Shaanxi Province, China: Journal of Air Force Medical University (2005). p. 1740.

[122] Zhang G . Observation on the therapeutic effect of treating 30 cases of viral enteritis in infants and young children with zhongjie feng. In: Orthopaedics. Wuhan, Hubei Province, China: Tongji Hospital Affiliated to Huazhong University of Science and Technology p. 212. doi:10.1001/archpedi.1934.01960090129014

[123] Wang G . Observation on the efficacy of sarcandra injection combined with anisodamine in the treatment of autumn diarrhea in children. In: Chinese community doctors. Beijing, China: Jilin Northeast Asia Publishing and Media Group Co., Ltd. p. 26.

[124] Ding L Tian L Du D . Observation on the therapeutic effect of zhongjiefeng injection combined with simida in the treatment of acute infantile rotavirus enteritis. J Handan Med Coll. (2005) 5:420–1.

[125] Han T Zhang Y Zheng G Guo Y . From pathogenic mechanisms to therapeutic perspectives: A review of gut microbiota and intestinal mucosal immunity in inflammatory bowel disease. Front Immunol. (2025) 16:1704651. doi:10.3389/fimmu.2025.1704651 41306971 PMC12643992

[126] Li Y Zhang Y . Randomized controlled study of compound zhuangjiefeng aerosol in the treatment of chronic pharyngitis. Electronic J Gen Stomatology. (2018) 5:190–191, 193. doi:10.16269/j.cnki.cn11-9337/r.2018.33.113

[127] Liao L Zhang Y . Clinical study on compound zhongjiefeng aerosol inhalation combined with point injection for acute pharyngitis of wind-heat type. New Chin Med. (2019) 51:210–2. doi:10.13457/j.cnki.jncm.2019.03.064

[128] Li N . Research on the preventive and therapeutic effect of camellia oil on gastric ulcer and its mechanism. In: Beijing institute of technology. Beijing, China: Beijing Institute of Technology. doi:10.1016/s0737-0806(99)80131-2

[129] Hu T . Research on the anti-gastric ulcer action mechanism of kangfuxin liquid. In: Chengdu university of traditional chinese medicine. Chengdu, China: Chengdu University of Traditional Chinese Medicine (2018).

[130] Xie Z Xie N . Clinical observation on the treatment of ulcer with zhongjiefeng capsule combined with omeprazole. J Med Forum. (2009) 30:77–8.

[131] Hou J Li M . Clinical observation on the treatment of chronic atrophic gastritis with sarcandra scabra. Shanghai Med Pharm J. (2009) 30:283–5.

[132] Huang Y . Efficacy of zhong-jie-feng in the triple therapy for the eradication of helicobacterpylori. Shanghai Med Pharm J. (2000) 12:12–3.

[133] Wang Y . Observation on the therapeutic effect of zhongjie feng in patients with gastric ulcers. Electronic J Clin Med Literature. (2019) 6:12. doi:10.16281/j.cnki.jocml.2019.80.010

[134] Wang Q Wu W . Application of zhongjiefeng in treating stomach ulcer and ulcer diameter. Clin J Chin Med. (2019) 11:35–7. doi: 10.3969/j.issn.1674-7860.2019.14.012

[135] Wang G Chen D Lin R . Advances in studies on chemical constituents and its quality control of whole plant of Sarcandra glabra. Chin Traditional Herbal Drugs. (2003) 8:109–11.

[136] Du M . Antibacterial traditional chinese medicine zhongjie feng. Shanghai Med Pharm J. (2007) 9:416. doi:10.2165/00128415-201214100-00121

[137] Li X Wang D Zeng S Xiong X . The determination of trace elements in sarcardra glabra (thunb)nakai and the summary of their functions in physiological process. Environ Exploitation. (1997) 4:7–9.

[138] Cong S Bi W Jiang L . Treatment of advanced non-small cell lung cancer with zhongjiefeng injection combined with chemotherapy. Chin J Cancer Prev Treat. (2005) 2:156. doi:10.16073/j.cnki.cjcpt.2005.02.029

[139] Huang D Huang H Lu Y . Clinical observation on the treatment of locally advanced nasopharyngeal carcinoma with combined chemoradiation and radiotherapy. Chin J Integrated Traditional Western Med. (2013) 33:456–8. 23841261

[140] Huang Y Zhang Y . Zhuangjiefeng injection combined with radiotherapy in the adjuvant treatment of nasopharyngeal carcinoma. Chin J For Clin. (2005) 6:63.

[141] Tang C Qin J Wang R . Clinical observation on the anti-radiation-induced oxidative damage of fengshui extract from zhongjie. Int J Lab Med. (2008) 29:883–884, 887.

[142] Qin Y Wang R Ma S Lv J Kang M . Observation on the clinical efficacy of zhujiefeng combined with pf regimen in the treatment of nasopharyngeal carcinoma. Guangxi Med J. (2013) 35:1317–1318, 1326. doi: 10.11675/j.issn.0253-4304.2013.10.14

[143] Dong Y Zhang L Shi L Du J Xie B . Clinical efficacy of zhongjiefeng injection in the treatment of patients with advanced esophageal cancer and its effect on the serum vegf and s100a4 levels. Prog Modern Biomedicine. (2019) 19:3712–3715, 3634. doi:10.13241/j.cnki.pmb.2019.19.026

[144] Clinical study on zhongjiefeng injection combined with apatinib in treatment of advanced gastric cancer. Drugs Clinic. (2017) 32:1114–7. doi:10.11569/wcjd.v24.i5.759

[145] Li Q Zheng L Hao Z Du M . Effect of zhongjiefeng tablets on the immune function of patients with colorectal cancer undergoing postoperative chemotherapy. Shanghai Med Pharm J. (2008) 9:420–2.

[146] Toxicological study of Sarcandra glabra. J Guizhou Med Univ. (1998) 1:43–4. doi:10.19367/j.cnki.1000-2707.1998.01.015

